# Phylogenomic analysis of the *Porphyromonas gingivalis - Porphyromonas gulae* duo: approaches to the origin of periodontitis

**DOI:** 10.3389/fmicb.2023.1226166

**Published:** 2023-07-19

**Authors:** Mauricio Morales-Olavarría, Josefa Nuñez-Belmar, Dámariz González, Emiliano Vicencio, Jaime Andres Rivas-Pardo, Cristian Cortez, Juan P. Cárdenas

**Affiliations:** ^1^Centro de Genómica y Bioinformática, Facultad de Ciencias, Ingeniería y Tecnología, Universidad Mayor, Santiago, Chile; ^2^Escuela de Tecnología Médica, Facultad de Ciencias, Pontificia Universidad Católica de Valparaíso, Valparaíso, Chile; ^3^Escuela de Biotecnología, Facultad de Ciencias, Ingeniería y Tecnología, Universidad Mayor, Santiago, Chile

**Keywords:** *Porphyromonas gingivalis*, virulence factors, phylogenomics, orthogroups, dN/dS, Tajima D value, gene gain/loss model

## Abstract

*Porphyromonas gingivalis* is an oral human pathogen associated with the onset and progression of periodontitis, a chronic immune-inflammatory disease characterized by the destruction of the teeth-supporting tissue. *P. gingivalis* belongs to the genus *Porphyromonas,* which is characterized by being composed of Gram-negative, asaccharolytic, non-spore-forming, non-motile, obligatory anaerobic species, inhabiting niches such as the oral cavity, urogenital tract, gastrointestinal tract and infected wound from different mammals including humans. Among the *Porphyromonas* genus, *P. gingivalis* stands out for its specificity in colonizing the human oral cavity and its keystone pathogen role in periodontitis pathogenesis. To understand the evolutionary process behind *P. gingivalis* in the context of the *Pophyoromonas* genus, in this study, we performed a comparative genomics study with publicly available *Porphyromonas* genomes, focused on four main objectives: (A) to confirm the phylogenetic position of *P. gingivalis* in the *Porphyromonas* genus by phylogenomic analysis; (B) the definition and comparison of the pangenomes of *P. gingivalis* and its relative *P. gulae*; and (C) the evaluation of the gene family gain/loss events during the divergence of *P. gingivalis* and *P. gulae*; (D) the evaluation of the evolutionary pressure (represented by the calculation of Tajima-D values and dN/dS ratios) comparing gene families of *P. gingivalis* and *P. gulae*. Our analysis found 84 high-quality assemblies representing *P. gingivalis* and 14 *P. gulae* strains (from a total of 233 *Porphyromonas* genomes). Phylogenomic analysis confirmed that *P. gingivalis* and *P. gulae* are highly related lineages, close to *P. loveana*. Both organisms harbored open pangenomes, with a strong core-to-accessory ratio for housekeeping genes and a negative ratio for unknown function genes. Our analyses also characterized the gene set differentiating *P. gulae* from *P. gingivalis*, mainly associated with unknown functions. Relevant virulence factors, such as the FimA, Mfa1, and the hemagglutinins, are conserved in *P. gulae, P. gingivalis,* and *P. loveana*, suggesting that the origin of those factors occurred previous to the *P. gulae* - *P. gingivalis* divergence. These results suggest an unexpected evolutionary relationship between the *P. gulae - P. gingivalis* duo and *P. loveana*, showing more clues about the origin of the role of those organisms in periodontitis.

## 1. Introduction

The *Porphyromonas* genus comprises Gram-negative, asaccharolytic, non-spore-forming, non-motile, and obligate anaerobic species (Paster et al., 1994; Summanen and Finegold, 2015). Its ecological niche is mainly the oral cavity of humans and other mammals, therefore being described by some authors as indigenous to the oral microbiome. It has also been isolated from the urogenital and gastrointestinal tracts and infected wound sites in humans and other mammals (Summanen et al., 2009; Sakamoto and Ohkuma, 2013; Rajilić-Stojanović and de Vos, 2014; Sakamoto et al., 2015; Sato et al., 2015; Zamora-Cintas et al., 2018; Guilloux et al., 2021). There are several officially described *Porphyromonas* species (Gibson and Genco, 2006). Of these species, *Porphyromonas gingivalis* is one of the most studied because of its key role in the pathogenesis of human periodontitis. It is a chronic, non-communicable, immuno-inflammatory disease characterized by the irreversible destruction of dental supporting tissues, collectively known as the periodontium. Periodontitis is caused by the constant challenge of the dysbiotic subgingival biofilm, where *P. gingivalis* acts as a keystone pathogen. Therefore, the uncontrolled immune response against these pathogens is responsible for the destructive nature of this disease (Hajishengallis and Lamont, 2014).

Due to its prevalent preference for the oral cavity, *Porphyromonas* evolution could be influenced by niche construction, a process in which the organisms periodically change the local resource distribution in ways that enhance their own fitness. As a keystone pathogen, the large impact on the structure and function of microbial communities can be influenced by perturbations in the microenvironment caused by niche construction, with a concomitant effect on the evolution of itself and the community (Laland et al., 1999). For example, *P. gingivalis* is closely related to *P. gulae*, a member of the oral microbiome of various mammalian hosts (Fournier et al., 2001); additionally, some virulence factors found in *P. gingivalis*, such as the fimbrial proteins, are also present in *P. gulae,* showing high sequence conservation (Hamada et al., 2008; Fujiwara-Takahashi et al., 2020).

*P. gingivalis,* as a key member of the dysbiotic subgingival microbiota, is capable to colonize, invade, and destroy tooth-supporting tissues (periodontium) via the expression of several virulence factors (Hajishengallis and Lamont, 2014; Zenobia and Hajishengallis, 2015). Despite having a relatively low abundance in the subgingival biofilm, this pathogen has keystone abilities that cause microbial dysbiosis and alter the host’s immune response, promoting the pathologic destructive inflammatory environment and potentially influencing the course of other inflammatory disorders, even causing systemic complications (Olsen et al., 2017).

This pathogen synthesizes, expresses, and secretes several virulence factors contributing to its pathogenesis, including lipopolysaccharide (LPS), gingipains, hemagglutinins, and fimbriae, among others (Nakayama and Ohara, 2017). All those factors contribute to the damage of periodontal tissue and exacerbate inflammatory responses. For instance, LPS released after bacterial lysis and secreted through outer membrane vesicles (OMVs) activates Toll-like receptor 4 (TLR4) (Waller et al., 2016), promoting destructive periodontal inflammation (Zhang D. et al., 2015). The cysteine protease family members, the gingipains, play crucial roles in complex host-pathogen interactions. In multispecies heterotypic communities, its protease activity degrades ligands linked to interspecific aggregation and coaggregation, influencing the structure and composition of biofilms (Jia et al., 2019). Moreover, the degradation of host proteins as a pathogenicity strategy enables the bacterium to evade the immune response (Chen et al., 2023), modulate signaling pathways that increase its virulence (Lourbakos et al., 2001), and acquire essential nutrients during infection (Guo et al., 2010). Additionally, fimbriae play a key role in host tissue adhesion (Chen et al., 2023) as well as bacterial aggregation and coaggregation, two processes necessary for the formation of oral biofilms (Hasegawa and Nagano, 2021). The two main fimbriae systems in *P. gingivalis,* FimA, and Mfa1, are involved in the interaction with the host-epithelial surface, the immune response, autoaggregation, and the aggregation of other oral bacteria, such as *Treponema* spp. and *Streptococcus gordonii* (Chung et al., 2000; Yamada et al., 2005; Hajishengallis et al., 2006; Harokopakis et al., 2006; Eskan et al., 2007; Kuboniwa et al., 2009). The *fim* and *mfa* gene clusters contain a diversity of polymorphisms that could be associated with different genotypes and are often utilized to classify *P. gingivalis* strains (Hasegawa and Nagano, 2021). In the case of periodontitis-like diseases in other mammals, *P. gulae* adhesion systems have been reported as key players in the development of periodontitis; moreover, *P. gulae* can invade human gingival epithelial cells *in vitro*, and its efficiency could be associated with the *fimA* genotype (Inaba et al., 2019). In cats, types B and C fimbriae were frequently detected in subjects with periodontitis, which increased the use of *fimA* as a marker related to virulences in periodontitis (Iwashita et al., 2019).

The current knowledge available indicates that there are several *Porphyromonas* species and that only a few species are involved in periodontal disease, being *P. gingivalis* and *P. gulae*. The availability of genomic information for different *Porphyromonas* strains and metagenome-assembled genomes (MAGs) raises the opportunity for the genomic analysis of several aspects of evolution and development of the pathogenic properties of *P. gingivalis* in the context of its genus and its close relatedness with *P. gulae*. Despite there are some genomic studies comparing *P. gingivalis* strains in order to make associations between genetic content and its pathogenic properties (Chen et al., 2004; Lenzo et al., 2016; Chen et al., 2017; Romero-Lastra et al., 2017; Mendez et al., 2019), currently, there are only a few studies covering possible relationships between evolution and gene content for *P. gingivalis* in comparison with other microorganisms. One remarkable study performed with 32 isolates from 18 species (O’Flynn et al., 2015) showed that *P. gingivalis* genomes contained some distinctive features, such as genes involved in iron transport and heme utilization, whereas lacked other genes (involved in processes such as protoporphyrin biosynthesis), when they were compared with sequences from other *Porphyromonas* species. Another study (Endo et al., 2015) compared *P. gingivalis* genomes with the sequences from other members of the so-called “red complex” (*Treponema denticola* and *Tannerella forsythia*), a set of organisms involved in the development of periodontal disease (Mohanty et al., 2019); this study predicted metabolic complementations in the fatty acid biosynthesis pathways between those organisms, suggesting a potential effect of pathway complementation in the development of their roles in disease.

Despite the availability of previous studies and genomic information, the relationship between genetic diversity, pathogenesis, and evolution of the adaptation in *P. gingivalis* to its role in human periodontitis remains to be clarified. Considering the potential evolutive history of *P. gingivalis* in the context of its genus, and the existence of other species such as *P. gulae* exhibiting similar characteristics in terms of niche and mechanism of infection in other mammals, a comparative genomics study with an evolutive perspective can help to understand how *P. gingivalis* acquired its features to be a keystone pathogen. Since virulence is the product of complex pathogen-host interactions (Diard and Hardt, 2017); the evolutionary analysis of virulence factors could help to explain the acquirement of the lifestyle of a keystone pathogen, is an important milestone to understand the evolution of some members of *Porphyromonas*. In addition to virulence factors, other accessory proteins in the machinery of *Porphyromonas* could show evidence of adaptation to the oral microbiome. Understanding the evolutionary process behind the evolution of *P. gingivalis* as a keystone pathogen could give signs of how other *Porphyromonas* had evolved in a different way to potentially become a keystone pathogen, like potentially could be *P. gulae*. In this study, we describe *Porphyromonas* pangenome and explore the phylogenetic relation of *P. gingivalis* virulence with other members of the genus, especially *P. gulae*, the closest species to *P. gingivalis*.

## 2. Methodology

### 2.1. Genome dataset definition

*Porphyromonas* genomes were selected from NCBI Genbank FTP site (August 2022). The dataset was filtered using two criteria: their taxonomic genomic identity and degree of completeness and contamination. The taxonomic identity was established by using the program ‘*classify_wf*’ of the GTDB-TK program, version 2.1.0 (Chaumeil et al., 2019), using the database release 207 as the reference, selecting all genomes classified into the *Porphyromonas* genus (“g__*Porphyromonas*” or “g__*Porphyromonas*_A”). Genome completeness and contamination were calculated using the program ‘*lineage_wf*’ from CheckM version 1.1.3 (Parks et al., 2015); only those genomes with completeness equal to or higher than 90%, and contamination below 5%, were selected, as previously suggested for “high-quality drafts” (Bowers et al., 2017).

### 2.2. Definition of species groups

In order to detect the genomic species represented among the selected genomes, we combined the prediction from GTDB-TK (see above) with the prediction of clusters defined by average nucleotide identity (ANI) values. All genomes were compared in an all-*vs*-all manner using FastANI version 1.32 (Jain et al., 2018) with default parameters. The raw pairwise comparison data was filtered, discarding all ANI values below 95%, the classical intra-species boundary for microbial genomes (Richter and Rosselló-Móra, 2009). Filtered pairwise comparisons were analyzed by the *MCL* program, creating putative genomic species clusters as observed in network clustering (van Dongen and Abreu-Goodger, 2012).

### 2.3. Annotation, identification of orthogroups, and phylogenomic tree

All members of the final dataset were annotated *de novo* using Prokka, version 1.11 (Seemann, 2014) (relevant parameters: *-metagenome -kingdom Bacteria -addgenes*). Orthogroups from the set of the final dataset were calculated by Orthofinder version 2.5.5 (Emms and Kelly, 2019) with the ‘*-og*’ parameter. For phylogenomic tree elaboration, a concatenated multiple sequence alignment was constructed from a set of 38 single-copy conserved orthogroups by using MAFFT v. 7.490 (parameters: *-maxiterate 1,000 -localpair*) (Katoh et al., 2019). The alignment was used by *iqtree* v. 2.1.4 (Nguyen et al., 2015) (parameters: *-m TEST -alrt 1,000*) to generate a maximum likelihood-based tree with an aLRT with 1,000 replicates as the branch support test. The phylogenomic tree was visualized using the *Toytree* python package (Eaton, 2020), or with the tool FigTree v.1.4.4^1^ when it corresponds. The genomic information from *Tannerella forsythia* (assembly GCA_000238215.1) was utilized as the outgroup for the *Porphyomonas* genus tree due to its relatedness with the genus (Summanen and Finegold, 2015).

### 2.4. Pangenome analyses and definition

The pangenome represents the collection of all groups of orthologous genes (orthogroups) from a set of genomes (Moldovan and Gelfand, 2018). To analyze the pangenome of *P. gingivalis* and *P. gulae*, separated Orthofinder executions were performed with the proteomes from each cluster without any outgroup (parameters: *-M msa -y*). The orthogroup matrix (including unassigned orthogroups) was obtained for each run and utilized for different pangenome metrics. Pangenome curves were created using the *panplots* function in R (created by *SioStef*),^2^ using 1,000 permutations. The *alpha* values for Heap’s law equation were calculated using the *curve_fit* function of the *scipy* package in Python, using as objective the equation “a * (x ** b).” The determination of shell, cloud, “soft-core” and core components of the pangenome was deduced from the complete orthogroup matrix by using Python scripts with the *pandas* package, considering *core* gene families as the orthogroups present in 100% of the strains, soft-core groups as present in between 90 and 99.9% of the strains, shell as groups present between 89 and 15% of strains, and cloud as the gene families present in between 14% and the equivalent to two strains. Unique groups can be deduced from the set of “species-specific orthogroups,” and the “unassigned genes,” both reported by Orthofinder. Figures were created with *ggplot2* and the *ggarrangment* R packages.

### 2.5. Predictive proteomic analysis

For predicted proteomes was made by combining the prediction of EggNOG mapper version 2.1.6 (Cantalapiedra et al., 2021) (parameters: “--tax_scope_mode narrowest --tax_scope prokaryota_broad --go_evidence experimental”).

### 2.6. Gene gain/loss model for *Porphyromonas gingivalis* - *Porphyromonas gulae* divergence tree

In order to create a general gene gain/loss model, all proteomes from genomes from *P. gingivalis* and *P. gulae*, in addition to one genome of *P. loveana* as the outgroup were compared using Orthofinder as previously mentioned. The generated orthogroup matrix was utilized for the generation of a phylogenomic tree. This tree and the binary version of the complete orthogroup matrix were used by the software Count (Csurös, 2010) for the calculation of gene gain/loss rates following the Wagner parsimony model, using convergence criteria were set to a likelihood delta of 0.05 with a maximum of 1,000 rounds, and the same penalty score (equal to 1) for gains and losses. The final tree was represented using the ETE3 Python package (Huerta-Cepas et al., 2016).

### 2.7. Evolutionary metrics: Tajima D pairwise dN/dS ratios and selection among sites

Tajima’s D value is a statistical test that infers rates of rare alleles and assigns scores to the orthogroups, therefore, detecting variation from a neutral model of molecular evolution (Zhang Q. et al., 2015). In order to calculate Tajima’s D value, the nucleotide sequences from the coding sequences of the single-copy conserved orthogroups of *P. gingivalis*-only or *P. gulae*-only, when corresponding, were utilized. Sequences were aligned with MAFFT, as mentioned above. Nucleotide multiple alignments were utilized to calculate Tajima’s D values using the *tajima.test* function from *pegas* R package.^3^ Graphs were created with *ggplot2*.

Since that dN/dS is more suitable for comparisons between sequences with considerable distances (Rocha et al., 2006), dN/dS values were computed from the nucleotide sequence of the single-copy orthogroups conserved in *both P. gingivalis and P. gulae* genomes, using *P. loveana* as outgroup for both comparisons. Each orthogroup set was aligned using MAFFT (G-INS-i mode); calculation of dN, dS, and ω was made by using CODEML program from the PAML package(Yang, 2007), using the following parameters: “*runmode = −2, seqtype = 1, CodonFreq = 3, model = 1, NSsites = 0, icode = 0, fix_kappa = 0, kappa = 1, fix_omega = 0, omega = 0.5*.” Pairwise comparisons with distances equal to zero, dN/dS > 5, and dS > 10 were discarded from the analysis. For all pairwise comparisons, the statistical significance among groups was evaluated using the Kolmogorov–Smirnov (*ks.test* in R) test (value of *p* <0.01).

### 2.8. Phylogenetic analyses of virulence factors of *Porphyromonas*

Hidden Markov Model (HMM) profile search (using HMMER 3.1b) were performed for the following Pfam entries to search for the following virulence factors: PF06321 (“*P_gingi_FimA,”* detectable in both FimA and FimC proteins), PF15495 (“*Major fimbrial subunit protein type IV, Fimbrillin, C-terminal,”* found in Mfa1 proteins), PF07675 (“*Cleaved Adhesin Domain”*; present in both gingipains and hemagglutinins), PF10365 (*DUF2436*, as a confirmatory hit for gingipains and hemagglutinins), PF01364 (“*Peptidase family C25,”* found in gingipains but not in hemagglutinins), and PF04371 (“*Porphyromonas-type peptidyl-arginine deiminase”*), found in the peptidyl-arginine deiminase (PAD) enzyme associated with protein citrullination in *P. gingivalis* (Gabarrini et al., 2015). Proteins from the *Porphyromonas* dataset were retrieved and aligned with MAFFT (G-INS-i mode). Phylogenetic trees of each PFAM were inferred using *iqtree* v. 2.1.4 (Nguyen et al., 2015) with the maximum likelihood (ML) method with an aLRT with 1,000 replicates as the branch support test (main parameters: *-m TEST --alrt 1,000*). For tree visualization, the FigTree v.1.4.4 software (see text footnote 1) was utilized.

## 3. Results

### 3.1. An up-to-date phylogeny of the *Porphyromonas* genus and representation of species groups

**Figure 1:**
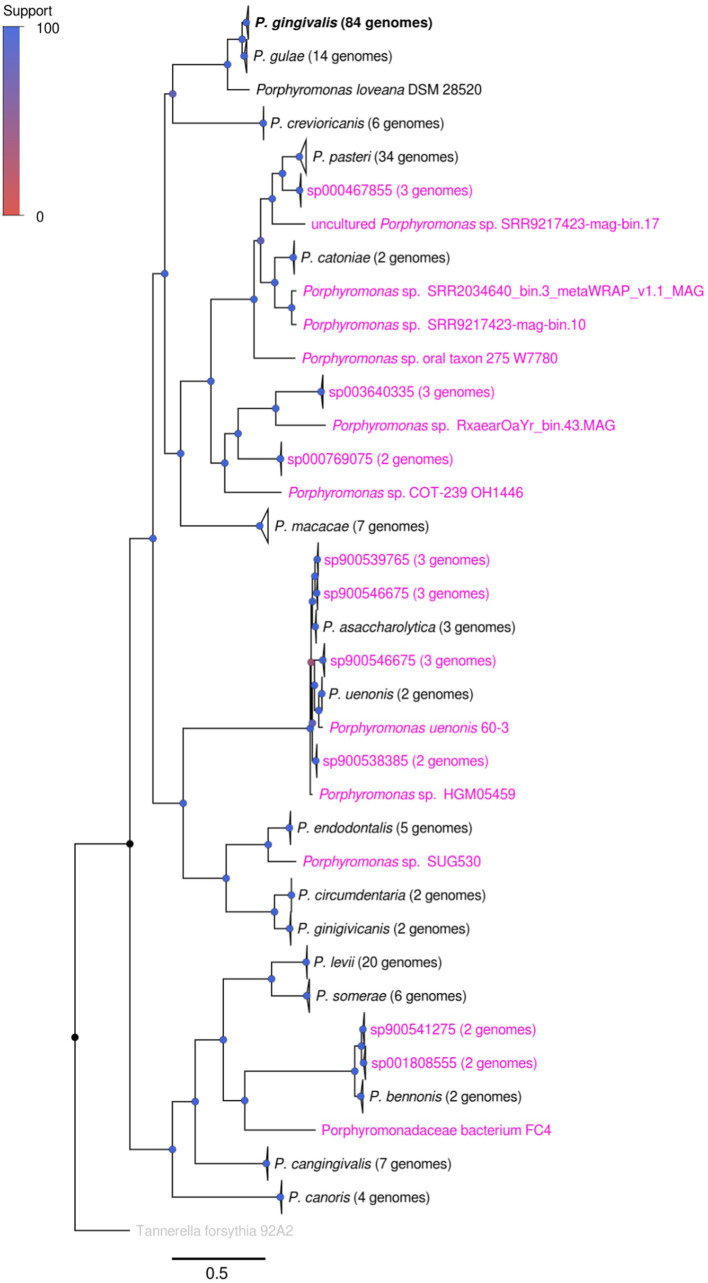
Phylogenomic tree showing the relationship between different *Porphyromonas* genomic species. The tree was created from the alignment of 38 single-copy conserved protein families, using the maximum likelihood method in IQTREE, with the use of the approximate likelihood ratio test (aLRT) as the branch support test. Taxa names colored in magenta represented genomes or species groups without an official name classification, according to GTDB-TK and manual review (see Supplementary Table S2); taxa names in black represented species groups with an official name. Branch support is represented by colors in each node, according to the *Support* color scale.

The *Porphyromonas* genus comprises a set of several organisms from both human and animal sources (Summanen and Finegold, 2015). Using the content available in the NCBI Genbank Genome repository, with the additional filter using taxonomic and completeness/contamination standards, the *Porphyromonas* dataset utilized in this study comprises 233 genomes, representing 36 genomic species clusters (Figure 1; Supplementary Figure S1; Supplementary Tables S1, S2). The five most represented genomic species in the dataset were *P. gingivalis*, *P. pasteri*, *P. levii*, *P. gulae*, *P. macacae* and *P. cangingivalis*, with 84, 34, 20, 14, 7, and 7 genomes, respectively. Of the set of genomic species, 19 clusters do not have an official species name; in most of those cases, only the GTDB-TK assignment was shown. Moreover, some strains, such as *Porphyromonas* sp. SUG530 (GCA_022772085.1), uncultured *Porphyromonas* sp. SRR2034640 _bin.3_metaWRAP_v1.1_MAG (GCA_915070105.1) or uncultured *Porphyromonas* sp. RxaearOaYr_bin.43.MAG (GCA_943912835.1), do not contain any species-level classification in GTDB-TK (Supplementary Table S1). The *P. uenonis* 60–3 strain (isolated from a vaginal sample) is part of a different species cluster than the canonical *P. uenonis* (DSM 23387, isolated from a sacral decubitus ulcer (Finegold et al., 2004)). This latter finding suggests that strain 60–3 could correspond to an undescribed species. The undescribed species (taxa with magenta names in Figure 1) cover an important fraction of the *Porphyromonas* tree, suggesting an extensive uncharacterized genetic content among the genus.

This phylogenomic tree, made from 38 conserved orthogroups (Figure 1), confirmed several previously reported phylogenetic associations between different members of the *Porphyromonas* genus. For example, the closed relationship between *P. gingivalis* and *P. gulae* has been previously shown in different studies (Fournier et al., 2001). Interestingly, this study also found a strong relatedness between the *P. gulae - P. gingivalis* duo with P. *loveana*, an organism isolated from the oral cavity of a musky rat kangaroo, a marsupial (Bird et al., 2016). In addition to this finding, the close relationship between *P. uenonis* and *P. asaccharolytica* was also shown previously (Finegold et al., 2004), as well as the close relationship between *P. levii* and *P. somerae* (Summanen et al., 2009). However, from now on, this study will be focused on the genome set of 84 *P. gingivalis* and 14 *P. gulae* strains.

### 3.2. Comparing the pangenomes from *Porphyromonas gingivalis* and *Porphyromonas gulae*

**Figure 2:**
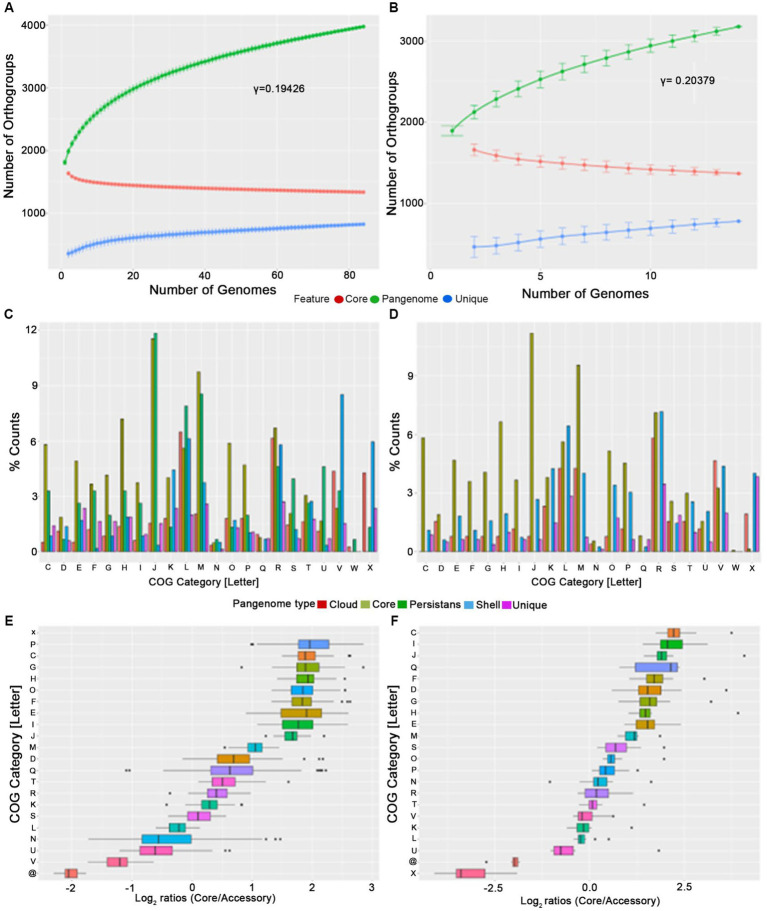
Pangenome analysis of *P. gingivalis* **(A,C,E)** and *P. gulae* **(B,D,F)**. The analysis included pangenome accumulation curves **(A,B)**, a barplot of the relative percentage of counts for each COG category **(C,D)**, and the Log2 ratios of the percentage of core vs. accessory genes for each category **(E,F)**.

Both the mutually highly related *P. gingivalis* and *P. gulae* are involved in periodontal diseases in their respective hosts (Lenzo et al., 2016). Therefore, studying evolutionary relationships between those organisms could be important to analyze to explain the origin of the *P. gingivalis* phenotype. Given that we found 84 high-quality genomes for *P. gingivalis* and 14 for *P. gulae*, we first compared them by analyzing each pangenome separately. For this purpose, the Orthofinder output was used to generate a gene matrix to calculate core, “persistent,” shell, cloud, and unique gene families, as well as to calculate the pangenome accumulation curves (Figures 2A,B; Table 1). Additionally, the percentages of different COG categories (assigned to each gene by using EggNOG mapper) were plotted among the different fractions (Figures 2C,D), and the Log2 of the ratios between the Core and the “Accessory” (the combination of “persistent,” shell, cloud, and unique gene fractions) among the different genomes were represented into boxplots (Figures 2E,F). The pangenome accumulation curves, fitted to the Power Law equation (Figures 2C,D), suggest that both pangenomes are open (𝛾 > 0, see Table 1), a feature that may reflect the contact of *P. gingivalis* and *P. gulae* with several other microorganisms in a complex environment (Rouli et al., 2015), as the mammalian oral cavity. Comparing the different functional COG categories suggests that categories associated with biosynthesis and metabolism (represented by letters C to H) are more represented in the Core and persistent fractions in both *P. gingivalis* and *P. gulae* pangenomes. Moreover, the calculation of the Log2 ratios between the Core and “Accessory” fractions for both pangenomes (Figures 2E,F) showed that categories C, G, H, I, J, and M (representing the COG categories “Energy production and conversion,” “Carbohydrate transport and metabolism,” “Coenzyme transport and metabolism,” “Lipid transport and metabolism,” “Translation, ribosomal structure and biogenesis,” and “Cell wall/membrane/envelope biogenesis,” respectively) have a median Log2 (Core/Accessory) higher than 1. In the case of the *P. gingivalis* pangenome, categories P (“Inorganic ion transport and metabolism”), O (“Posttranslational modification, protein turnover, chaperones”), and E (“Amino acid transport and metabolism”) have also higher Log2(Core/Accessory) ratios. COG categories with higher Log2 (Core/Accessory) ratios in *P. gulae* only were D (“Cell cycle control, cell division, chromosome partitioning”), F (“Nucleotide transport and metabolism”), and Q (“Secondary metabolites biosynthesis, transport and catabolism”). In all those cases, the comparison between the Core and Accessory groups was significant (*p* < 0.01, two-sided Wilcoxon Rank Sum test in R). These results suggest that, in general, housekeeping and central metabolism/ transport functions were more represented in the Core than in the Accessory gene set. This feature has been observed in other studies with other bacterial models (Gaba et al., 2020; Hyun et al., 2022). In contrast, genes without any COG hit (marked as “@” category in Figure 2) were most prevalent in the accessory fraction of the pangenome in both *P. gingivalis* and *P. gulae*. In the case of *P. gulae*, category X (“Mobilome: prophages, transposons”) was also more represented in the accessory than in the core gene fraction of the pangenome. In *P. gingivalis*, genes from category V (“Defense mechanisms”) had more negative Log2(Core/Accessory) ratios, whereas category X was not suitable for Log2 calculation, since the Core fraction had zero genes in that category (Figure 2C). It is worth noticing that differences from data from categories X, V, and without COG between Core and Accessory groups were significant as well (*p*< 0.01, two-sided Wilcoxon Rank Sum test).

**Table 1:** Main statistics from pangenome analysis for *P. gingivalis* and*P. gulae.*

	*P. gingivalis*	*P. gulae*
Total genomes	84	14
Total Pangenome Genes	3,976	3,179
Core genes (in 100% of genomes)	1,335	1,367
Soft-core genes (<100%, more than 95%)	145	0
Shell genes (<95%, more than 15%)	550	786
Cloud genes (<15%, at least in two genomes)	1,124	247
Unique genes (in just one genome)	822	779
gamma value (power law equation)	0.19426	0.20379

**Figure 3:**
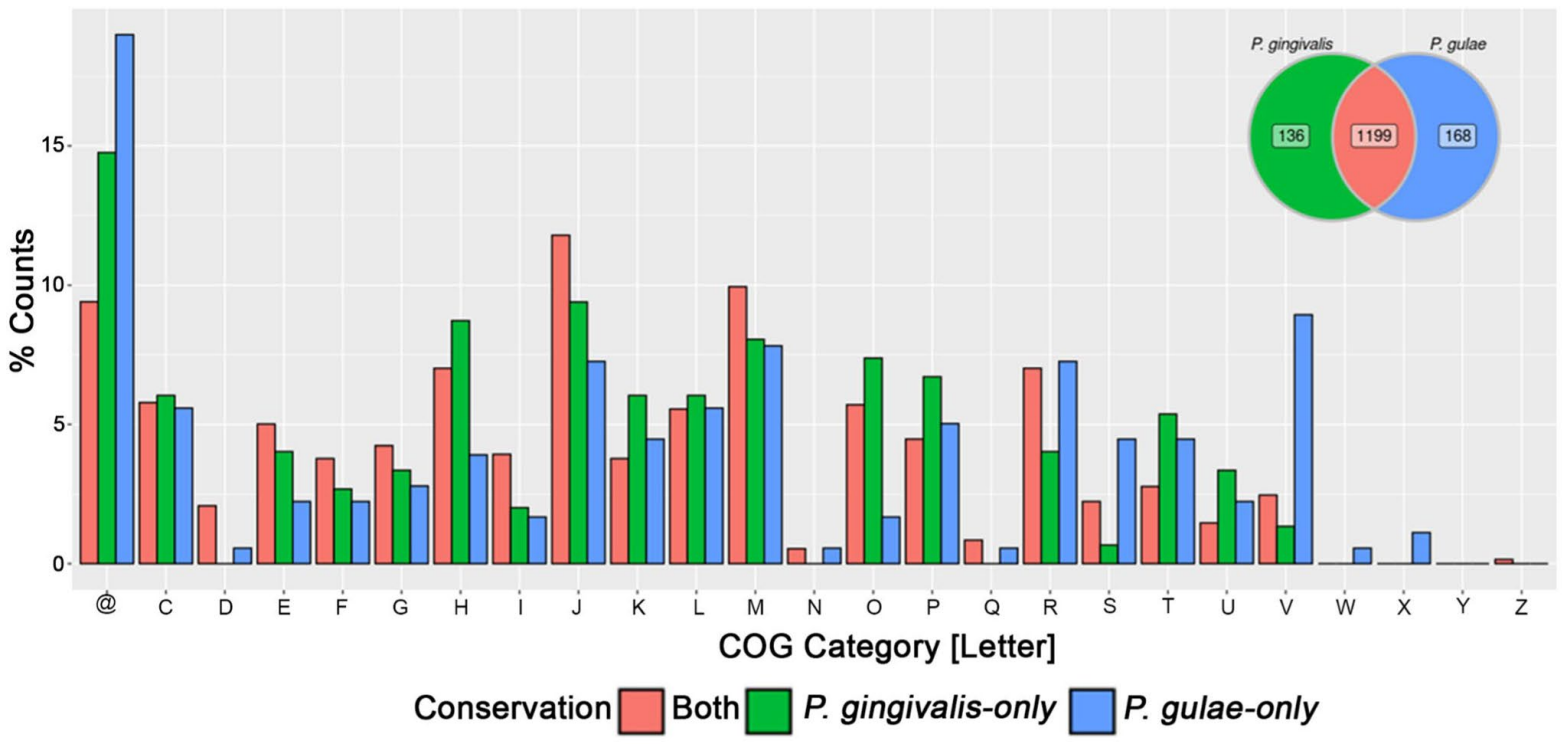
Comparison of the pangenome core of *P. gingivalis* and *P. gulae*. Bidirectional hits between the pangenome cores of each organism were represented in the Venn diagram (upper right), as well as the gene representatives without bidirectional hits in either *P. gingivalis* or *P. gulae*. The barplot of the relative percentage of counts for each COG category represented the genes found in the core from both organisms or found only in each pangenome core. @: Genes without any COG.

The core gene fractions from *P. gingivalis* and *P. gulae* contained common and differentiative features. In order to compare them to define which functions were exclusive to the core fractions in each organism, a bidirectional comparison was performed with representatives from each gene from the core between those two species (Figure 3). This comparison showed a set of 1,199 bidirectional hits, and a total of 136 and 168 genes without a clear bidirectional hit in the core pangenome components of *P. gingivalis* and *P. gulae*. Respectively. Among the functional categories observed in the common and differential core contents, two cases are worth noticing between *P. gingivalis* and *P. gulae*. First, in the first one, there are more genes associated with category V (“Defense mechanisms”) in *P. gulae* than in *P. gingivalis*; some of those functions are associated with a Cas-Cmr CRISPR system, several genes for multidrug efflux pump AcrA/B proteins and ABC-type antimicrobial peptide transport system, permease component (SalY)- encoding genes. In counterpart, categories H and O (“Coenzyme transport and metabolism” and “Posttranslational modification, protein turnover, chaperones,” respectively) are more present in the core of *P. gingivalis* than in *P. gulae*; some of those genes encode for ABC systems for siderophores, a set of serine proteases and a cation-transporting P-type ATPase (data not shown).

### 3.3. Gene gain/loss model in the *Porphyromonas gingivalis*/*Porphyromonas gulae* ancestry

**Figure 4:**
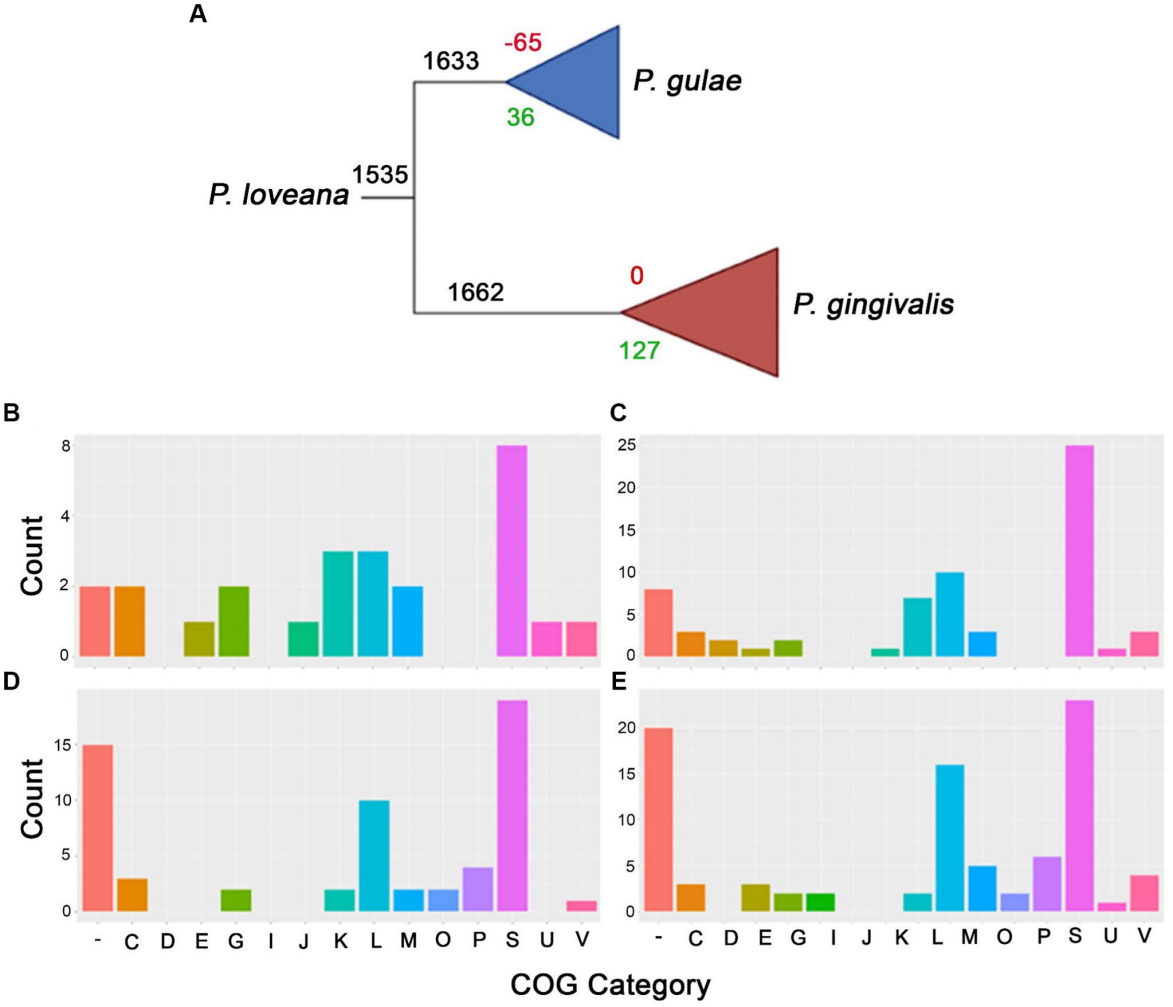
Gain and loss model genes representation for *P. gingivalis* and *P. gulae* comparison. **(A)** Collapsed phylogenetic tree representing the total amount of genes for each branch (black), as well as the gain (green) and loss (red) genes. The bottom panel represents the COGs assignment for each branch in the collapsed tree, i.e., gained and missed genes for *P. gulae* ancestor [**(B,C)**, respectively], and the set of gained and missed genes for *P. gingivalis* ancestor [**(D,E)**, respectively].

Our phylogenomic analysis of the *Porphyromonas* genus confirmed that *P. gingivalis* and *P. gulae* are strongly related, as well as that *P. loveana* is also directly related to them (Figure 1). In order to check about the genetic gain and loss events during divergence between *P. gingivalis* and *P. gulae*, the orthogroup and phylogenetic information from 84 *P. gingivalis* and 14 *P. gulae* genomes (including one *P. loveana* genome as an outgroup) were used to create a gene gain-loss model to represent the number of genes present in the predicted common ancestors across the phylogeny, by using the tool Count with Wagner parsimony analysis. The gain-loss model tree for this set of genomes is shown in Figure 4A (detailed version in Supplementary Figure S2), where each node has the number of genes analyzed, as well as the number of genes gained and lost. A set of 1,662 gene families is predicted in the ancestor of the divergence between *P. gulae* and *P. gingivalis* lineages; in this context, the last common ancestor (LCA) of all 84 *P. gingivalis* genomes contained 1,661 genes, involving the loss of 95 genes and the acquisition of 94 genes in comparison with the previous ancestor. In the LCA of *P. gulae*, 1,633 genes were found in the predicted ancestor, with a gain of 35 genes and a loss of 65 genes. Across the intraspecific branches in the *P. gulae/P. gingivalis* tree, relative gene gain/loss events were relatively low, occurring with a higher frequency of gain and loss events when nodes are closer to the terminal leaves (Supplementary Figure S2).

In order to understand which classes of functions were acquired in the differentiation process between *P. gingivalis* and *P. gulae*, the functions of the genes found in the LCAs of all *P. gingivalis* and/or *P. gulae* were annotated with EggNOG mapper using COGs (Clusters of Orthologous Genes) categories at the *root* level. Considering the predicted contents of the LCAs for *P. gingivalis* and *P. gulae*, the next step is to define the content acquired and missed during the formation of those predicted ancestors. According to COGs assignments, the gained genes in the LCA for *P. gulae* were mainly associated with “K” and “L” categories (Figures 4B,C), related to “Transcription” and “Replication, recombination, and repair,” with 0.18 and 0.18%, respectively. However, this LCA also suffered the loss of several genes involved in the “L” category (0.61%), and in other more generic categories such as “S” (“Function unknown”), as well as genes without any category. Some categories, such as “E” (Amino acid transport and metabolism), were more acquired in this predicted ancestor. Some of the categories with only loss were “O” and “P” (Posttranslational modification, protein turnover, chaperones, and Inorganic ion transport and metabolism), with one and four genes.

All the 94 acquired genes in the LCA of *P. gingivalis* belonged to the Unknown function category (Figures 4D,E); on the other hand, the missed genes with no COG category in this node corresponded to 20 OGs. As well as *P. gulae* “K” and “L” were the won categories with high values (0.42 and 0.60%). Interestingly, category “E” (related to amino acids) has more OGs loss compared to those won it and with *P. gulae.* Some categories with only won are the “D” (Cell cycle control, cell division, chromosome partitioning) and “J” (Translation, ribosomal structure, and biogenesis). As the *P. gulae* LCA, categories “O” and “P,” have been lost (0.12 and 0.36%).

A BlastP analysis against SwissProt database for the genes acquired by *P. gulae* and *P. gingivalis* LCAs was performed to obtain more clues about the function of those genes. Results showed that 34 out of 36 proteins for *P. gulae* and 85 out of 94 for *P. gingivalis,* were predicted as hypothetical proteins. Global inspections of this protein and their functions for *P. gulae* (data not shown) show that 19 of those 34 sequences were hypothetical proteins; the rest corresponded to genes associated with secretion systems, transcriptional regulators, post-translational modifications, enzymes involved in metabolism, unknown protein domains, mobilome proteins, and DNA interaction proteins. For *P. gingivalis,* 46 of those 85 genes were hypothetical proteins; the rest encoded genes associated with secretion systems, transcriptional regulation, post-translational modifications, stress response and pathogenicity, DNA plasmid partition, and phage interaction.

### 3.4. Evolutive pressure in the shared core orthogroups from *Porphyromonas gingivalis* and *Porphyromonas gulae*

The presence of shared genes in the *P. gulae* and *P. gingivalis* lineages, raises the question about the potential effect of natural selection in the different species. In order to evaluate this possibility, the shared orthogroups of *P. gingivalis* and *P. gulae* were calculated (via Orthofinder) using the 99 genomes from these organisms (and the *P. loveana* genomes as the outgroup) and the common single-copy core orthogroups were utilized for the calculation of the evolutionary selection parameters (dN, dS, t, and dN/dS ratio).

**Figure 5:**
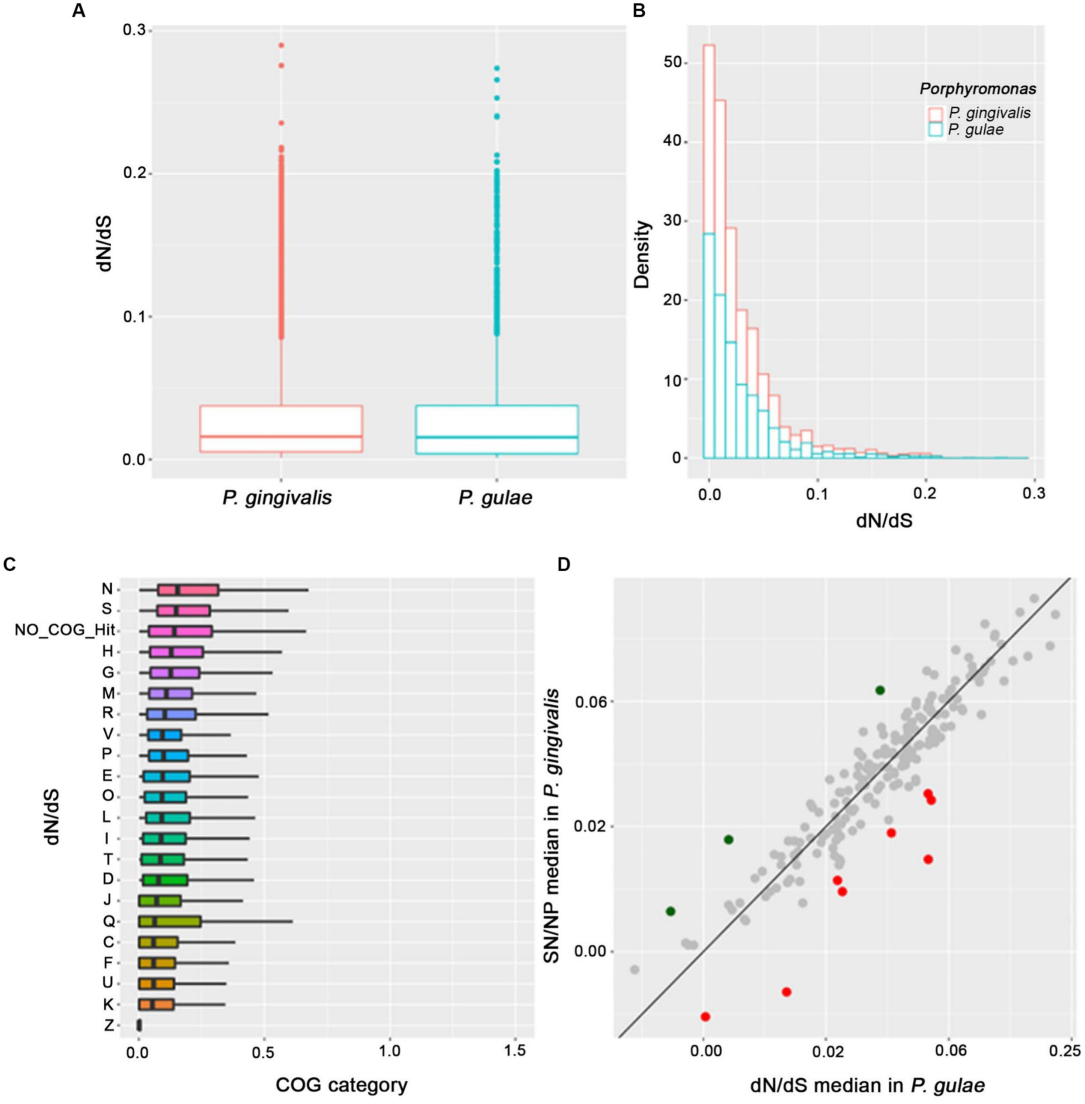
Analysis of proteome content evaluated under natural selection and the COGs category assignments. The main focus was put on the *P. gingivalis* and *P. gulae* comparison, for each omega ratio value **(A)**, the distribution of the omega value **(B)**, the distribution of each COGs and their respective values of omega ratio (ordered by the median) for each protein in the proteome **(C)** and the comparison of the omega value median of each OG (shown in gray for those without positive selection, as well as green and red for those selected as positive in *P. gingivalis* and *P. gulae,* respectively) in the comparison *P. gingivalis* versus *P. gulae* to detect signals of positive natural selection **(D)**.

The distribution of the dN/dS ratio between *P. gingivalis* and *P. gulae* showed an expected behavior of a long proportion of genes with dN/dS < 1 (Figures 5A,B), which is a signal of purifying selection in most of the genes. The mean for the dN/dS ratio was 0.028, compared to the mean of 15.185 of dS, showing a higher rate of synonyms mutations in the OGs set (data not shown for dS alone version and Figure 5). The means of the ratios between *P. gingivalis* and *P. gulae* are similar (0.0282 and 0.0273, respectively, with no significative difference according to the Kolmogorov–Smirnov test), however, in the same way, the mean of the dS between the two species are similar (15 and 16.1, respectively), this result shows that most of the orthogroups between both species do not have strong evidence of positive selection. If the comparisons are divided by the functional category of their genes, COGs categories with the highest dN/dS ratio medians can be found (Figure 5C); the analysis of the pairwise distributions showed that the N and S COG categories (“Cell motility” and “Unknown function,” respectively) harbored the highest median for comparisons. By contrast, K and Z categories (“Transcription” and “Cytoskeleton,” respectively) possessed the lower medians for dN/dS values.

Considering this feature, we perform an additional analysis where each orthogroup is compared between their values for the *P. gingivalis - P. loveana* and *P. gulae - P. loveana* pairwise calculations. In Figure 5C, the scatterplot represents the median of each orthogroup considering their values for the *P. gingivalis - P. loveana* and *P. gulae - P. loveana* calculations. This comparison found 11 orthogroups with differentially higher ratios (3 for *P. gingivalis* and 9 for *P. gulae*). Using a Blastp analysis versus the SwissProt database, a more detailed description of each orthogroup was obtained (Table 2). Those genes harbored different functions, including a variety of housekeeping functions. For example, in *P. gingivalis* those genes were related to anaerobic respiration, glycogen storage, and DNA damage recognition, as 4-hydroxybutyryl-CoA dehydratase/vinyl acetyl-CoA-Delta-isomerase, glycogen synthase and Uvr ABC system protein B, respectively. On the other hand, *P. gulae* has protein related to cell wall maintenance, ion membrane translocation, anaerobic respiration, signal recognition receptor, as well as amino acid, and fatty acid metabolism.

**Table 2:** List of orthogroups found under positive selection with their respective protein function association in the comparison between *P. gingivalis* and *P. gulae*.

Species feature	OG	Function
Higher in *P. gingivalis*	OG0000556	4-hydroxybutyryl-CoA dehydratase/vinylacetyl-CoA-Delta-isomerase
Higher in *P. gulae*	OG0000576	D-alanine--D-alanine ligase
Higher in *P. gulae*	OG0000350	Ion-translocating oxidoreductase complex subunit G
Higher in *P. gulae*	OG0000351	Na(+)-translocating ferredoxin:NAD(+) oxidoreductase complex subunit E
Higher in *P. gingivalis*	OG0000702	Glycogen synthase
Higher in *P. gulae*	OG0000267	Signal recognition particle receptor
Higher in *P. gulae*	OG0000713	Acetoacetyl-CoA:acetate/butyrate CoA transferase alpha subunit
Higher in *P. gingivalis*	OG0000394	UvrABC system protein B
Higher in *P. gulae*	OG0000720	Acyl-CoA dehydrogenase, short-chain specific

### 3.5. Tajima values for *Porphyromonas gingivalis* and *Porphyromonas gulae* core orthogroups

**Figure 6:**
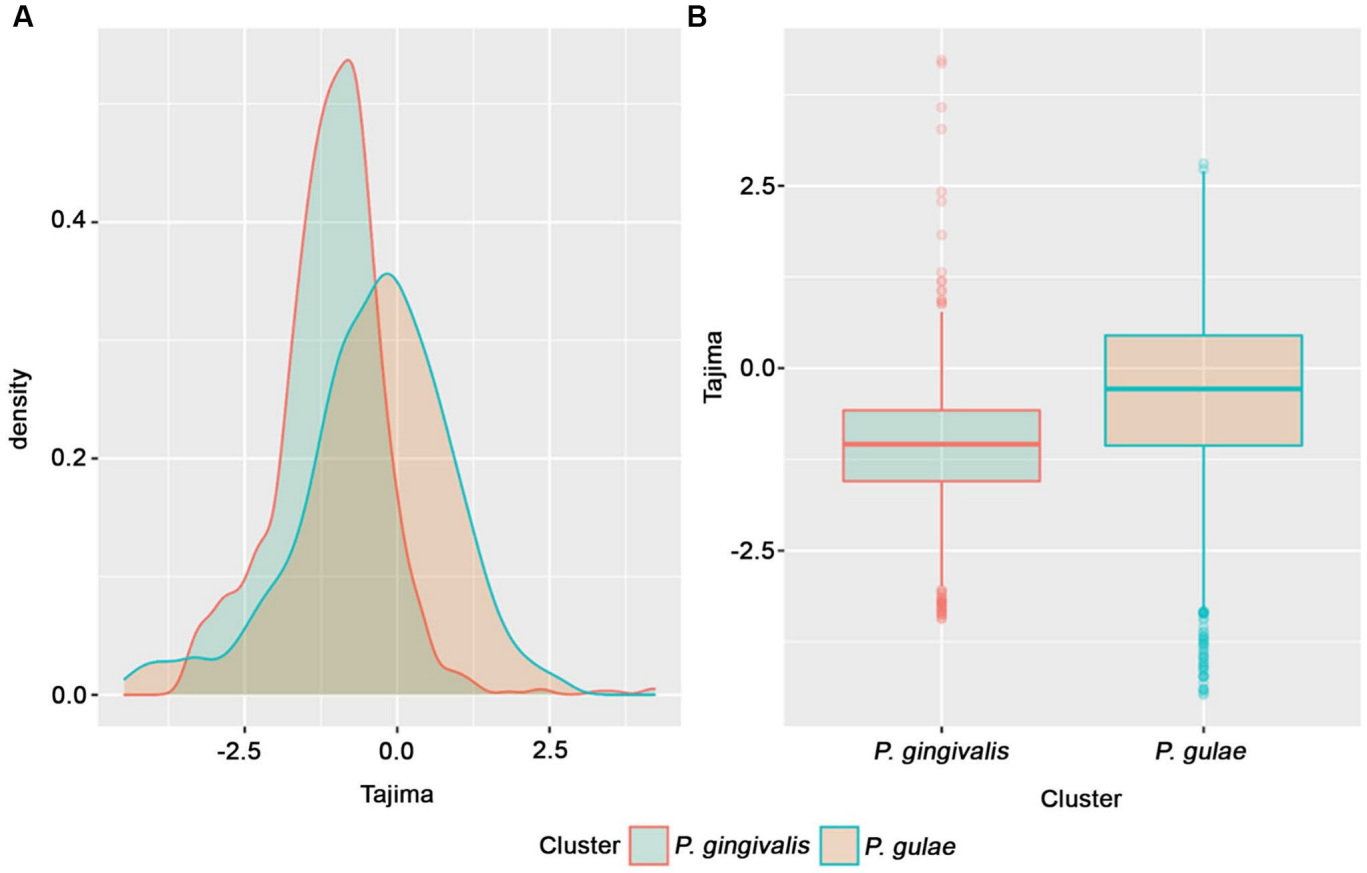
Tajima’s D value distributions for *P. gingivalis* and *P. gulae* core gene families. **(A)** histogram showing density distributions for Tajima values data for *P. gingivalis* and *P. gulae*; **(B)** boxplots for Tajima values clustered by species.

Tajima D value is a statistical test that uses DNA data to detect natural selection (Korneliussen et al., 2013). In order to detect general trends in the conserved CDS in both *P. gingivalis* or *P. gulae*, the set of single-copy core genes obtained from the previously analyzed *P. gingivalis* and *P. gulae* pangenomes separately, we performed the test with 1,335 core families of *P. gingivalis* and 1,367 core families from *P. gulae*. Figure 6A shows the histogram curves densities of D value for two core orthogroups. *P. gingivalis* has a peak between 0 and − 1.5. The negative values are associated with an excess of rare variation, which is interpreted as population growth or positive selection (Carlson et al., 2005). *P. gulae* Tajima’s value distribution is more shifted to the negative but closer to 0 than *P. gingivalis*. Overall, *P. gingivalis* D-value distribution has a higher negative shift than genes from *P. gulae* and it was significant (*p*-value <2×10^−16^, Welch Two Sample *t*-test; Figure 6B). *P. gingivalis* may exhibit lower values either because of positive selection (more rare variants) or because a population is recovering or expanding after a bottleneck effect. The bottleneck effect refers to a reduction in the population size, resulting in the loss of genetic diversity of the species (Peery et al., 2012). In contrast, the more positive distribution of *P. gulae* values may reflect a possible population contraction or allele frequency stabilization associated with the emergence of this organism.

To observe these results in more detail, the top ten OGs with the highest and lowest Tajima values of *P. gingivalis* were retrieved (Table 3). The most positive Tajima value was 4,222; which corresponds to a gene that encodes a putative RagB, which is part of an outer membrane transporter protein that functions as a peptide importer (Madej et al., 2020). Other genes found encoded proteins with functions such as TonB-dependent receptor P3, putative tyrosine-protein kinase in *cps* locus, dipeptide and tripeptide permease B, UDP-glucose 6-dehydrogenase TuaD, Peptide chain release factor 2, Major fibrium tip subunit FimD, and polysaccharide biosynthesis/export protein, in addition with some hypothetical proteins. These sets of genes have the most neutral/balanced selection in *P. gingivalis*. They are involved in multiple cellular processes, like heme metabolism homeostasis, heme, iron, and amino acid uptake from the host, regulation, and transport of capsular polysaccharides, protein synthesis, and function of bacterial fimbriae.

**Table 3:** List of orthogroups covering the 10 highest Tajima values of in the *P. gingivalis* core, including their protein names and functions (*p* < 0.05).

Top position	OG	Tajima value	Protein	Function
1	OG0000294	4.2226932	Hypothetical protein (RagB)	RagB is an outer membrane transporter protein that functions as a peptide importer in *P. gingivalis*.
2	OG0000293	4.17820696	TonB-dependent receptor P3	Protein involved in the acquisition of essential nutrients, such as iron and heme, from the host
3	OG0000559	3.574256432	Hypothetical protein	Unknown
4	OG0001241	3.348000822	Hypothetical protein	Unknown
5	OG0000454	3.275760793	Putative tyrosine-protein kinase in cps region	The cps (capsular polysaccharide synthesis) region is responsible for the biosynthesis of the capsular polysaccharides
6	OG0001351	2.715270824	Dipeptide and tripeptide permease B	Protein involved in the uptake of peptides that are used by the bacterium for the synthesis of various virulence factors, such as gingipains
7	OG0000872	2.418843779	UDP-glucose 6-dehydrogenase TuaD	Biosynthesis of the bacterial cell surface polysaccharide
8	OG0000873	2.287357949	Peptide chain release factor 2	Termination of protein synthesis during bacterial translation
9	OG0001381	1.90044368	Major fimbrium tip subunit FimD	Assembly and function of the bacterial fimbriae
10	OG0000455	1.826818797	Hypothetical protein (polysaccharide biosynthesis/export protein)	Assembly of the polysaccharide chain into a repeating unit, as well as its transport across the cell membrane

On the other hand, the top ten orthogroups with the lowest Tajima’s D value (Table 4), are genes with an exacerbated proportion of rare alleles (values between −3.22 and − 3.095). The most negative one encodes a hypothetical protein, that matches (by blast analysis in the NCBI platform) with another TonB-dependent receptor, which is involved in iron and heme uptake from the host. The other proteins are: calcium-transporting ATPase 1, DUF4248 domain-containing protein, cAMP-activated global transcriptional regulator CRP, type VI-B CRISPR-associated RNA-guided ribonuclease Cas13b, 10 kDA chaperonin, transcriptional regulatory protein QseB, Acyl-CoA dehydrogenase C-terminal domain-containing protein, peptidoglycan-N-acetylglucosamine deacetylase, and GNAT family N-acetyltransferase. Their functions are related overall to iron and heme uptake from the host, calcium, and cAMP intracellular levels homeostasis, a prokaryotic defense mechanism against foreign genetic elements, regulation of biofilm formation, fatty acid metabolism, and cell wall modifications.

**Table 4:** List of orthogroups covering the 10 lowest Tajima values of in the *P. gingivalis* core, including their protein names and functions (*p* < 0.05).

Top position	OG	Tajima value	Protein	Function
1	OG0001008	−3.223375698	Hypoyhetical protein (TonB-dependent receptor)	Protein involved in the acquisition of essential nutrients, such as iron and heme, from the host
2	OG0000740	−3.211357054	Calcium-transporting ATPase 1	Ion pump involved in calcium homeostasis and tolerance to high concentrations of calcium
3	OG0000475	−3.206282293	Hypothetical protein (DUF4248 domain-containing protein)	Unknown, it is suggest that is involved in protein–protein interactions, DNA binding, or enzymatic activities
4	OG0001040	−3.204872637	cAMP-activated global transcriptional regulator CRP	Regulation of respond to chages in intracellular levels of the signaling molecule cyclic AMP (cAMP)
5	OG0000392	−3.186069821	Hypothetical protein (type VI-B CRISPR-associated RNA-guided ribonuclease Cas13b)	Prokaryotic defense mechanism against foreign genetic elements such as viruses and plasmids
6	OG0000511	−3.158209672	10 kDa chaperonin	Folding of newly synthesized proteins
7	OG0000636	−3.155269224	Transcriptional regulatory protein QseB	Response regulator that is activated by the QseC sensor kinase, involved in the regulation of biofilm formation and antibiotic resistance
8	OG0000667	−3.137577035	Hypothetical protein (Acyl-CoA dehydrogenase C-terminal domain-containing protein)	Catalyze the first step in the breakdown of fatty acids
9	OG0001389	−3.121169508	Peptidoglycan-N-acetylglucosamine deacetylase	Modification of peptidoglycan, an structural molecule of bacterial cell wall
10	OG0001157	−3.095009015	Hypothetical protein (GNAT family N-acetyltransferase)	Modification of a variety of cell wall components, including lipopolysaccharides and capsular polysaccharides

### 3.6. Virulence factor conservation and evolution among *Porphyromonas* genomes

*P. gingivalis* is known to produce a set of virulence factors involved in the formation of the periodontal disease (Aleksijević et al., 2022; Nuñez-Belmar et al., 2022). Among them, FimA and Mfa1 are filamentous structures essential for the interaction of *P. gingivalis* with other bacteria of the biofilm and with host cells (Nagano et al., 2018). *P. gulae* also was shown to exhibit these adhesive molecular systems (O’Flynn et al., 2015). The major and minor fimbriae exhibit adhesion properties that are suggested to play a central role in periodontitis development (Mendez et al., 2019). Other virulence factors are the gingipains and the Hemagglutinins (Hag). Hag proteins are bacterial surface glycoproteins associated with bacterial adhesion to erythrocytes and host cells (Mendez et al., 2019). For *P. gingivalis* metabolism the heme acquisition is essential, thus, hemagglutinins adhesive domain function is related to this process, facilitating the acquisition of heme. Hag-encoding genes (*hagA, hagB,* and *hagC*) are described as relevant virulence factors of *P. gingivalis* (Nakayama and Ohara, 2017). Gingipains are lysine- and arginine-specific proteinases secreted by *P. gingivalis*, and they are considered as one of the most important virulence factors in this organism; their principal function is the degradation of host proteins and the processing of the fimbriae subunits (Lunar Silva and Cascales, 2021). Finally, peptidyl-arginine deiminase (PDA) are enzymes previously reported in *P. gingivalis* capable of producing protein citrullination associated with their capabilities in microbe-host interaction and a role in some diseases such as arthritis (Maresz et al., 2013). The differential degree of presence/absence of orthogroups among Porphyromonads may also occur in the known virulence factors associated with *P. gingivalis* in periodontitis and other diseases. To check for other hits for a variety of the main virulence factors, a *hmmer* search for different Pfam domains (Supplementary Table S3) was performed, searching for families associated with previously reported virulence factors, with a posterior phylogenetic analysis of those hits. From the PFAM database, this analysis utilized the families identified as PF06321, containing sequences for FimA (genotypes I, II, and IV) and FimC; PF15495 containing Mfa1 and other related proteins; and PF010365, containing hemagglutinin and other proteins such as Lys-gingipains and Arg-gingipains.

**Figure 7:**
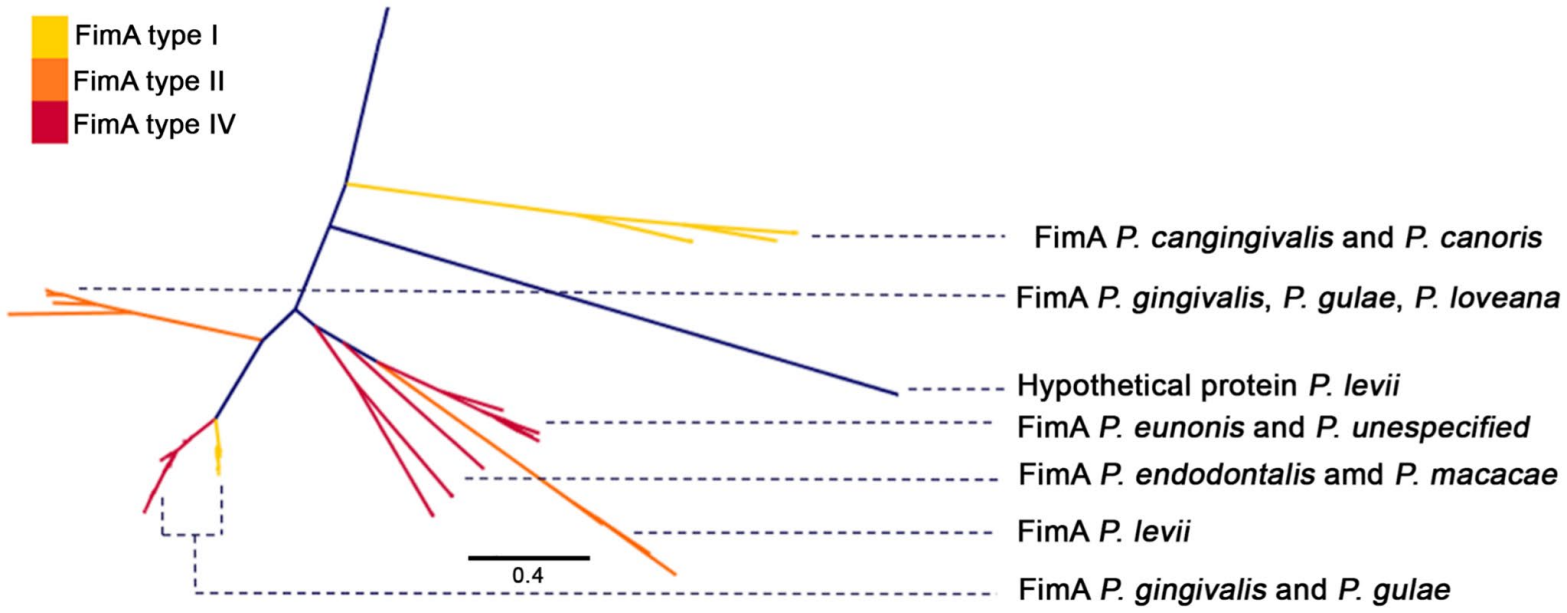
Phylogenetic tree, based on the FimA amino acid sequences (PFAM PF06321) of the *Porphyromonas* genus. FimA branches are colored according to FimA genotype (type I yellow, type II orange, and type IV red), and labeled with *Porphyromonas* species.

**Figure 8:**
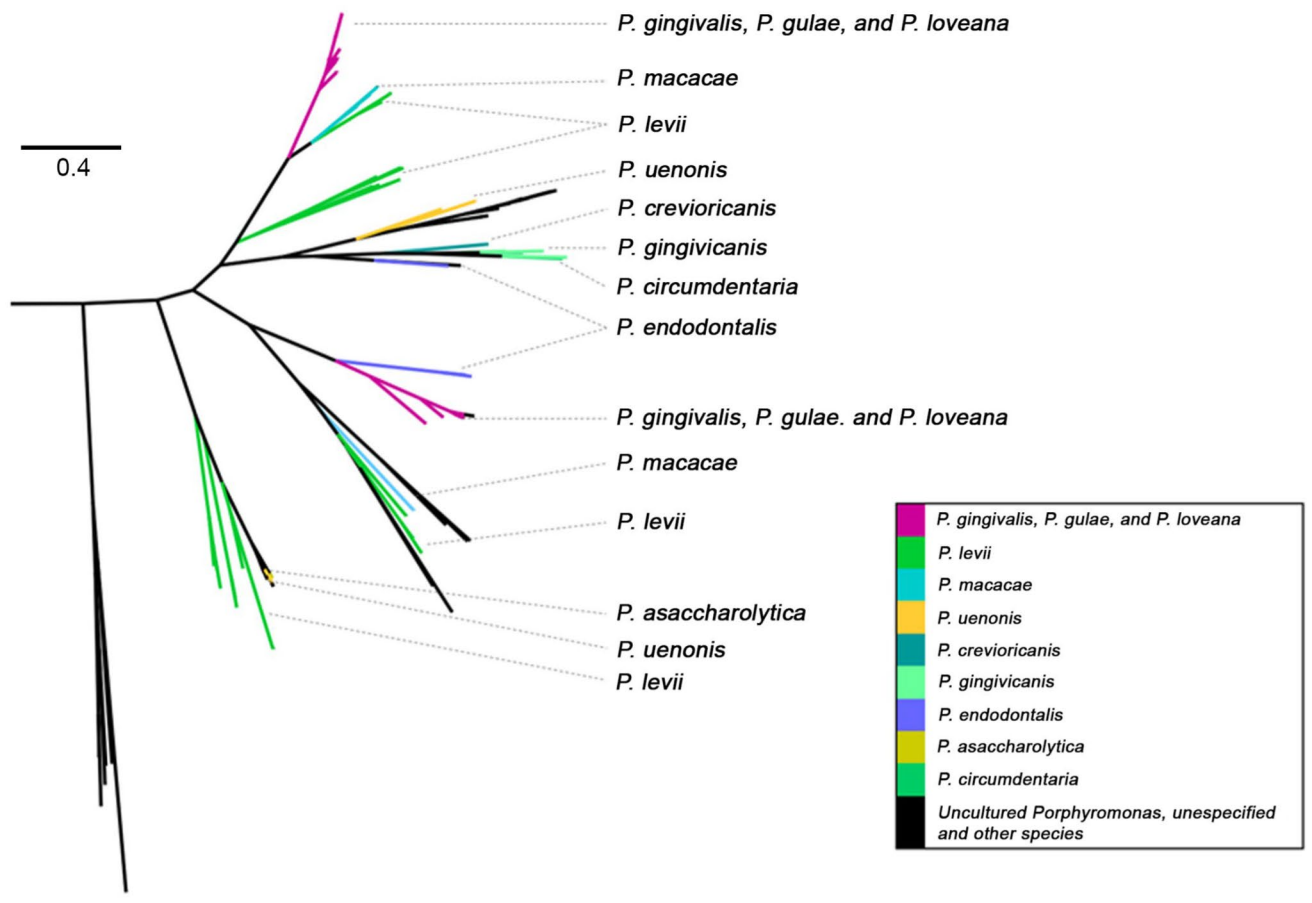
Phylogenetic tree, based on Mfa1 amino acid sequences (PFAM PF15495) of *Porphyromonas* genus. Tree branches were colored according to their taxonomic origin among *Porphyoromonas* species.

These analyses suggested that the number of genes containing several of those domains exhibited a differentiative pattern in the group of *P. gingivalis - P gulae - P. loveana*. For example, there are more genes containing the PF06321 domain (“*P_gingi_FimA”*) in those organisms than in the rest of the Porphyromonads. Hits for PF06321 and PF15495 (“*Major fimbrial subunit protein type IV, Fimbrillin, C-terminal”*) domains were widely distributed among *Porphyomonas* genomes, being detected in multiple copies in *P. gingivalis*, *P. gulae*, *P. loveana,* and *P. macacae*. A phylogenetic tree base on aminoacid sequences of FimA (Figure 7) and Mfa1 (Figure 8) from the selected *Porphyoromonas* genomes. The original PF06321 FimA phylogenetic tree (Supplementary Figure S3) showed two main branches: one of them represented the FimC (proteins from *P. gingivalis, P, gulae, P. loveana, P. levii*, and *P. crevioricaris*), and the second clade represented FimA from several Porphyromonads. The FimA clade is colored by each genotype (type I, type II, and type IV in Figure 7), and represented proteins from *P. cangingivalis, P. canoris, P. levii, P uenonis, P. endodontalis, P. macacae, P. levii, P. gingivalis, P. gulae,* and *P. loveana*. Genotype II FimA proteins were detected in the trifecta *P. gingivalis, P. gulae, and P. loveana*, whereas genotypes I and IV were found in *P. gingivalis* and *P. gulae*. Interestingly, FimA homologs were also detected in other members of the genus, such as *P. endodontalis, P. levii, P. cangingivalis, P. ueonis* and *P. canoris*; moreover, the most related to the ancestral branches were found in *P cangingivalis* and *P. canoris*, suggesting that this branch could predate the origin of the *P. gulae* - *P. gingivalis* duo.

The minor fimbriae Mfa1 was also present in a variety of species of the Porphyromonas genus (Figure 8), being detected in *P. gingivalis*, *P. gulae*, *P. loveana*, *P. macacae*, *P. levi*, *P. uenonis*, *P. crevioricanis*, *P. gingivicanis*, *P. circumdentaria*, *P. endodontalis*, *P. somerae*, and *P. asaccharolytica*. The original tree PF15495 phylogenetic tree (Supplementary Figure S4) showed two main branches, one of them clearly composed by Mfa1 homologs. In this subtree, two linages of *P. gingivalis* - *P. gulae* - *P. loveana* were detected: One is associated with *P. macacae* and *P. levi*, which each are isolated from dog oral cavity and cow skin lesions. The other lineage is associated with *P. endodontalis*, which is isolated from human subgingival biofilm and saliva, besides has been closely associated with periapical lesions of patients with periodontitis (Lombardo Bedran et al., 2012). This phylogenetic configuration suggests that, in *P. gulae* and *P. gingivalis*, the mfa1 genes could have two origins, dictated by their transfer from other members of the *Porphyromonas* genus.

**Figure 9:**
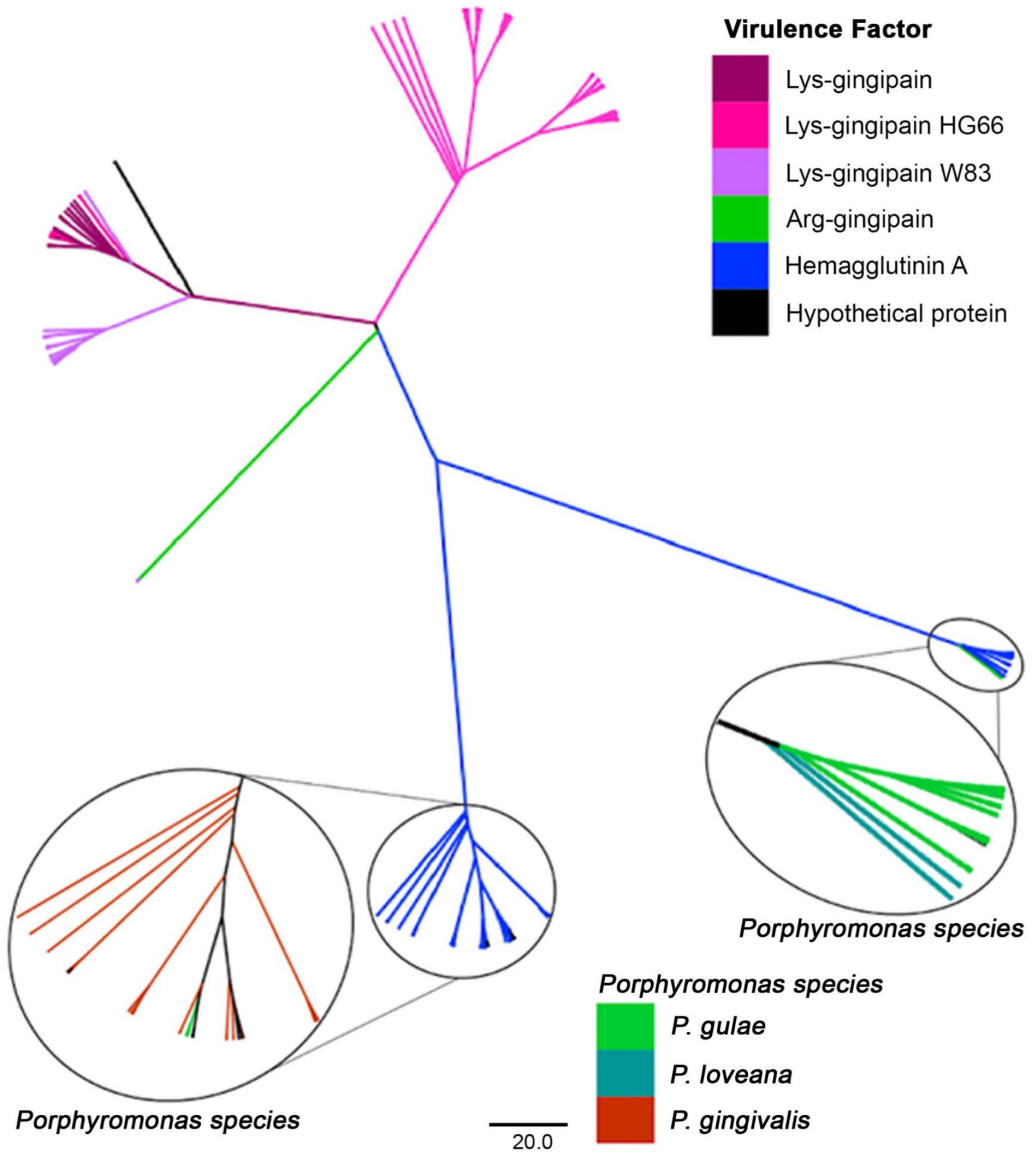
Phylogenetic tree, based on the hemagglutinin and gingipain amino acid sequences (PFAM PF010365) of *Porphyromonas* genus. Tree branches were colored according to their putative function, separating among hemaglutinins, Lys- or Arg-gingipains. Taxonomic origin is highlighted for the hemaglutinin clades.

The phylogenetic tree with protein sequences filtered by PFAM PF010365 from the *Porphyromonas* dataset (Figure 9) included proteins such as the hemagglutinins and the gingipains [divided into lysine-gingipains, arginine-gingipains, proteases with an adhesive hemagglutinin-like domain (Mysak et al., 2014)], as well as a set of hypothetical proteins. Lysine-gingipains are exclusively associated with *P. gingivalis*, whereas, the Arginine-gingipains were associated with *P. loveana* and *P. gingivalis*. The hemagglutinin A lineage was represented by two branches: one with *P. gulae* and *P. loveana* proteins, and the other with *P. gingivalis* and *P. gulae* proteins. The presence of hemagglutinins and gingipains in *P. loveana* may suggest that this species could have a similar pathogenic phenotype as observed in *P. gingivalis*.

For the case of the PAD proteins, a phylogenetic tree using those sequences showed that *Porphyromonas* PF04371 hits were distributed in two lineages (Supplementary Figure S5). One comprises the PAD proteins from *P. gingivalis*, *P. gulae,* and *P. loveana.* The other lineage contains the putative agmatine deiminases from nearly all members of the genus. This two-branch divergence suggests that the previous protein citrullination activity involved in pathogenic features in *P. gingivalis* could also be present in *P. gulae* and *P. loveana*.

## 4. Discussion

### 4.1. The uncovered diversity of the *Porphyromonas* genus and its relationship with the *Porphyromonas gingivalis* - *Porphyromonas gulae* duo

In order to investigate how *P. gingivalis* could become a pathogen in the context of the *Porphyromonas* genus, the first aspect we needed to analyze is to validate its position in relation with its relatives. We performed with a set of 233 high-quality *Porphyromonas* genomes a combination of a phylogenomic analysis with the use of genomic metrics for the establishment of genomic species clusters (Maturana and Cárdenas, 2021). The phylogenetic structure of the genus (Figure 1) showed that there are some clades mostly composed of undescribed species, most of them already classified by the GTDB (Supplementary Table S1). This feature has become common in several genera among bacterial and archaeal species as a reflection of their novelty in specialized databases (Chaumeil et al., 2022). Additionally, the phylogenomic analysis may propose new species or genera from previously described and undescribed strains (Sawana et al., 2014; Gupta et al., 2020). In this case, several undescribed *Porphyromonas* species could be representatives of different oral cavity inhabitants, as well as colonizers from other body sites, with their roles in host health and disease yet to be understood. For example, two undescribed species (mentioned as “oral taxon 278” and “oral taxon 275”) detected in plaque samples (Mei et al., 2020), and the misnamed strain 60–3 (named as a *P. uenonis* strain, despite it is not part of the species) may represent some relevant new species remaining to be officially named and described.

The phylogenomic analysis of the *Porphyromonas* genus confirmed the strong relatedness with *P. gulae* [formerly a *P. gingivalis* subspecies associated with non-human hosts, see (Fournier et al., 2001)], but also showed a strong relatedness with *P. loveana*, a strain isolated from the oral cavity of a musky rat kangaroo (Bird et al., 2016). The exact role of this latter species in oral health and disease in its host remains unclear. However, as seen in later results (see Section 3.6), several virulence factors were also conserved in this species, as well as the *P. gingivalis* - *P. gulae* duo. This may suggest a potential role in the periodontal disease of *P. loveana* in their marsupial host. This may also suggest two potential scenarios: (A) the divergence between *P. gingivalis/P. gulae* duo and *P. loveana* are the potential origins of the periodontal disease and it is as old as the divergence of early mammals and marsupials [estimated to occur between 130 and 170 million years ago (Cifelli and Davis, 2003)]; or (B) periodontal disease is potentially rooted in the mammals, regardless of the date of origin of the *Porphyromonas* species associated to it. To address these affirmations, it will be necessary to sequence more periodontitis-associated Porphyromonads in several other mammals.

### 4.2. Pangenome openness of *Porphyromonas gingivalis* and *Porphyromonas gulae* and the potential role of gained genes among LCAs during species divergence

Pangenome approaches allow us to know which genetic repertoire of a bacterial group, especially discover essential genes for the survival or even all the virulence factors in a specific bacterial species (Bosi et al., 2015; Rouli et al., 2015). Our analysis shows that the pangenome of *P. gingivalis* and *P. gulae* are in an openness stage (Figure 2; Table 1), suggesting that the gene content from both species will be accepting more new gene families while new genomes are added to the analysis. The genomic core for both organisms was enriched housekeeping functional categories whereas the accessory fraction of the pangenomes was enriched by genes without COG and associated with mobile elements and defense mechanisms. This adaptation of the accessory core in both species is a previously observed feature, reflecting a recent history of mobile element transfer and the utilization of fitness factors (Brockhurst et al., 2019). The presence of unknown genes Interestingly, some proteins present in the *P. gulae* were related to phage defense like CRISPR-Cas system associate proteins. Recent studies have suggested the presence of different phages in *P. gingivalis,* raising the potential for cross-phage infection between relative species like *P. gulae* and *P. gingivalis* (Matrishin et al., 2022).

The gene gain/loss analysis showed a comparable number of gene gain and gene loss events during the divergences between *P. gingivalis, P. gulae,* and *P. loveana*, with the number of gene gain/loss events after the speciation process. Since that previous evidence showed that selection could be a significant driver of gene loss and reductive genome evolution in bacterial species (Koskiniemi et al., 2012) and that gene content reconstruction can reflect the evolution of microbial pangenomes in a specific taxon (Iranzo et al., 2019; Maturana and Cárdenas, 2021), events observed during *P. gingivalis - P. gulae* bifurcation suggested a discrete role of gene gain/loss in this species delimitation, as a signal that those two species are very similar in the evolutionary pressure produced by their respective environments.

To our knowledge, there is no previous report about gain and/or loss events describing *Porphyromonas* evolution, even if it is focused only on *P. gingivalis*. The fact that several genes gained by the LCA of *P. gingivalis* were related to metabolic enzymes and transcriptional regulators, genes with these specific characteristics were categorized as essential for fitness in *P. gingivalis* (Miller et al., 2017). Moreover, one of the genes was involved in the nitro-groups (nitroreductase family protein). In the oral microenvironment, nitrosative stress could be challenging due to the high intake of dietary nitrate, some genes are necessary for the growth of this oral pathogen (Lewis et al., 2012). Some of the gained genes by both LCAs were related directly to the mobilome of this pathogen, which involved genes responsible for relaxase/mobilization nuclease, transposase, plasmid mobilization relaxosome, integrase, and transposons. All those elements play an important role in genetic rearrangement and are considered driving forces of bacterial diversification (Olsen et al., 2018). Two proteins that were identified as T9SS type A were gained in this node, T9SS translocates proteins to the outer membrane, which is important for virulence factors. These proteins play an important role in cell survival and fitness in response to the microenvironment (Lasica et al., 2017). One important virulence factor was reported, glycosyltransferase, which is important for capsular polysaccharide biosynthesis (Naito et al., 2008).

In the case of *P. gulae*, we found at least two type IX secretion systems which are interesting in the way that these kinds of systems are present in some bacteria species and could play two main roles, which are gliding motility or a weapon for some pathogens, as *P. gingivalis* described above. To our knowledge, this kind of system was not described previously in *P. gulae*, delving into the sequences inside the genome of some members of *Porphyromonas* could reveal some particular features. Proteins related to transcription factors process or interact with DNA as well as an energy process, where were pNresent between the genes gained by the *P. gulae* LCA. Some elements like relaxosome protein, which is a protein that allows the conjugation process, a type of horizontal transfer gene, this kind of system could play an important role in the genetic transfer of information between members of *Porphyromonas* (Watanabe et al., 2017). Finally, the LCA of *P. gulae*, like *P. gingivalis*, also gained a nitroreductase protein. These two species are members of the oral microbiome in mammals, as we described above the oral microenvironment is rich in nitro-groups.

### 4.3. Differential gene selection across *Porphyromonas gingivalis* and *Porphyromonas gulae* may be part of the specialization process

The analysis of dN/dS ratios is a method to study evolutionary pressure among macroevolution in gene families (Vitti et al., 2013). The measure of those values was performed for the shared core of *P. gingivalis* and *P. gulae*, considering *P. loveana* as the outgroup. The observation that the “N” and “S” categories contained the highest median for dN/dS data, could reflect a slightly higher diversification effect for genes associated with those categories; genes associated with category N were involved in functions such as cell cycle control, cell division, and chromosome partitioning, including proteins like ParA (Bignell and Thomas, 2001), a member of a large group of P-loop ATPases with a deviant Walker A motif, involved in DNA partitioning (Mishra and Srinivasan, 2022). Proteins like this could be under the effect of positive selection due to they are essential for proper DNA partitioning and cell division, in the same way, the effect of natural selection could give advantages to ensuring accurate DNA and improve fitness (Liu et al., 2008). Another protein found in category N was part of a complex involved in the translocation of lipopolysaccharide (LPS), the permease protein Lpt (Lpt). The translocation of LPS to the outer membrane could be related to cell motility and membrane dynamics (Veronika et al., 2011). LPS is an important virulence factor for *P. gingivalis* and/or *P. gulae*, the effect of positive selection could improve the impact of this virulence factor in the oral microbiome (Dashper et al., 2017). The S category is the second with the highest median; genes from this category were generally annotated as membrane proteins and lipoproteins. Further investigation is necessary to characterize those genes.

The *P. gulae* and *P. gingivalis* lineages could have different evolutionary pressures. The comparison of their respective values for the set of each shared orthogroup showed that only a few gene families contained a considerable signal making them candidates to have a more positive selection in one lineage than in another: three belonged to *P. gingivalis* and six to *P. gulae*. The function of some of these gene families could give some clues about the specialization of each lineage. For example, genes under positive selection in *P. gingivalis* were 4-hydroxy butyryl-CoA dehydratase/vinyl acetyl-CoA-Delta-isomerase, Glycogen synthase, and UvrABC system protein B. The first protein is involved in the oxidation of fatty acids mostly in anaerobic microorganisms, this protein has a relationship with *P. gingivalis*, *Clostridium aminobutyricum,* and *Archaeglobus fulgidus* (Gerhardt et al., 2000) and its positive selection may respond to the need to improve its fitness to synthesize lipids in their environment, in comparison with *P. gulae*. Glycogen synthesis could influence the survival mechanism of microorganisms (Park, 2015); previous studies in other pathogens such as *Vibrio cholerae* showed that glycogen biosynthesis is a fitness factor under nutrient-limiting conditions, such as the aquatic environment (Kamp et al., 2013). The UvrABC system protein B is part of a complex involved in DNA repair and with a relevant role in damage resistance in *P. gingivalis* during oxidative stress (Henry et al., 2012). The differentially higher positive selection may respond to the need to improve fitness by diversifying selection in those genes when the *P. gingivalis* lineage is compared with *P. gulae*.

### 4.4. Tajima’s D values suggest evidence of a bottleneck effect in the human host

As mentioned earlier, *P. gingivalis* and *P. gulae* are closely related phylogenetically (O’Flynn et al., 2015; Fujiwara-Takahashi et al., 2020). Additionally, the closest relative to them is *P. loveana*, a species isolated from the oral cavity of Australian marsupials (Bird et al., 2016). *P. gingivalis* and *P. gulae* emerged from a common ancestor, and although both are associated with periodontitis, they differ in host specificity. *P. gingivalis* is isolated from human samples and *P. gulae* from other mammal hosts (Fujiwara-Takahashi et al., 2020). Such different host specialties could be associated with changes in neutrality among core genes.

We performed the Tajima D statistical test with core gene families of *P. gingivalis* and *P. gulae* and generated curves and boxplots with all the Tajima’s scores for gene families of those organisms (Figure 6B). The differences among the Tajima values between species were significant (*p* < 2×10^−16^). *P. gingivalis* distribution presented a distribution towards lower Tajima’s D values than *P. gulae*., exhibiting less neutrality in their core orthologs (higher frequencies of rare alleles). This observation may be explained due to an expansion after a bottleneck effect.

The bottleneck effect refers to a past event that causes the reduction of the population since the outcome is the loss of genetic diversity, with impacts on the appearance of rare alleles (Peery et al., 2012). The foundation effect is caused when a small group of individuals establish a new population. This causes the emergence of a new population with less genetic diversity and an increment in the frequency of rare alleles (Nielsen, 2001). In the case of *P. gingivalis*, this may be explained due to its specialization as the only Porphyromonad causing periodontitis in the human oral cavity, exhibiting an important site, host, and activity specificity (Bostanci and Belibasakis, 2012). In contrast, its relative *P. gulae*, shows similar characteristics and a role in periodontal disease but is associated with a wider host range, as it is isolated from different mammals. Considering the pathogenicity and specificity of the hosts of both species, as well as the findings of our study, we dare to hypothesize that *P. gingivalis* experienced the bottleneck effect, followed by an amplification of rare alleles, during its colonization and establishment in the human oral cavity. We speculate that both *P. gingivalis* and *P. gulae*, from their same common ancestor, contained the virulence factors necessary for periodontal infection and after a speciation event, they evolved to adapt to their hosts, being *P. gingivalis* the one whose evolved to a different species in the human body. However, since the numbers of *P. gingivalis* and *P. gulae* genomes were unequal, sequencing of more *P. gulae* genomes and their posterior analysis can help to confirm these allele frequency differences.

### 4.5. Virulence factor conservation among *Porphyromonas* species shows a common pattern in lineage emergence for periodontal disease

Phylogenetic analyses for some of the most relevant virulence factors involved in periodontitis showed patterns that could be explained by the evolution of *Porphyromonas* species, as well as by their association with their hosts. For example, phylogenies for the major fimbriae (FimA) and the minor fimbriae (Mfa1) showed the co-clustering of *P. gulae, P. gingivalis* and *P. loveana* sequences, in agreement with the common evolutionary origin for those organisms. In the case of the different genotypes (I, II, and IV), the potentially closest clade to the root of the group is the type II genotype, which also includes the ortholog from *P. loveana*. The furthest branch contained FimA genotypes I and IV, encoded by *P. gingivalis,* and *P. gulae* strains. Current evidence has suggested that type I genotype is associated with samples from patients with periodontal health, whereas genotypes II and IV are associated with samples from patients diagnosed with periodontitis (Wang et al., 2020). This may suggest that the rise of the attenuated type I could be posterior to the emergence of the pathogenic type II, aspect that could ensure the early capability of *P. gingivalis* and *P. gulae* to succeed in their role in periodontal disease in their respective niches. In contrast, the late adaptation into genotypes I and IV may respond to dynamics of host specificity adaptations as long as *P. gulae* and *P. gingivalis* are adjusting to their new niches. Previous studies suggested that genotype IV fimbriae are associated to increased adhesion efficiency in comparison with genotype I (Mendez et al., 2019); this behavior may reflect a late adaptation to increase adhesion abilities after the formation of the “soft” genotype I phenotype.

The presence of FimA orthologs in other Porphyromonads (*P. cangingivalis, P. canoris, P. levii, P uenonis, P. endodontalis, P. macacae, P. levii*) expand the previous findings from (Fujiwara-Takahashi et al., (2020), which only considered *P. gulae* and *P. gingivalis*. In fact, one early clade in the FimA phylogeny contained orthologs from *P. cangingivalis* and *P. canoris*, species isolated from canine oral cavities. *P. cangingivalis* is isolated from health and early periodontitis-associated subgingival plaque, and *P. canoris* from subgingival plaque associated to periodontitis (Collins et al., 1994; Love et al., 1994). This finding suggests that the origin of FimA may predate the adaptation of the role of Porphyromonads in periodontal disease.

A similar “trifecta-enriched” phylogenetic pattern was also observed with Mfa1, a protein expressed by *P. gingivalis* on its surface. The primary function of the fimbrial systems is to adhere to host epithelial cells and colonize subgingival tissue. However, Mfa1 is also involved in microorganism auto-aggregation, microbe-microbe interactions, and suppression of the host immune response (Arjunan et al., 2016), via the evasion of autophagy-lysosome function of myeloid dendritic cell (DC) when it internalizes *P. gingivalis* (El-Awady et al., 2015). Mfa1 is also able to induce osteoclastogenesis by interacting with the Toll-like receptor of murine macrophage (RAW264 cells) *in vitro* (Suzuki et al., 2022). Osteoclastogenesis and consequent bone resorption is the hallmark of periodontitis (Hajishengallis and Lamont, 2016). Additionally, the *in vitro* interaction between *P. gingivalis* Mfa1 and the streptococcal antigen I/II receptor (SspA/B) of the early colonizer *Streptococcus gordonii* was demonstrated (Roky et al., 2020).

A phylogenetic tree of Mfa1 proteins was constructed from the PF15495 hits found in *Porphyromonas* genomes. Congruent Mfa1 homologs were detected in *P. gingivalis, P. gulae,* and *P. loveana,* in addition to *P. macacae, P. levi, P. uenonis, P. crevioricanis, P. gingivicanis, P. circumdentaria, P. endodontalis,* and *P. asaccharolytica*. Whereas *P. macacae* and *P. cangingivalis* were identified in the oral cavity of a variety of mammals (dogs, cats, monkeys, and bovines) (O’Flynn et al., 2015; Gabarrini et al., 2018). Additionally, *P. uenonis,* and *P. asaccharolytica* have been detected in the cervicovaginal tract and appendicitis- or peritonitis-associated samples (Finegold et al., 2004; Lithgow et al., 2022) or in diabetic patients with foot infections (Imirzalioglu et al., 2014). Another interesting aspect of the Mfa1 phylogeny is that homologs from the trio *P. gingivalis - P. gulae - P. loveana* are distributed in two separated clades suggesting that in those organisms, the origin of this protein was carried out twice. The separation of those clades is a resemblance of the existence of two proposed genotypes in the Mfa1 genes observed in *P. gingivalis* and *P. gulae*, the so-called 70-kDa and 53-kDa types (Fujiwara-Takahashi et al., 2020). Structural and more detailed evolutionary studies of those separated clades are worth a more profound next study.

**Figure 10:**
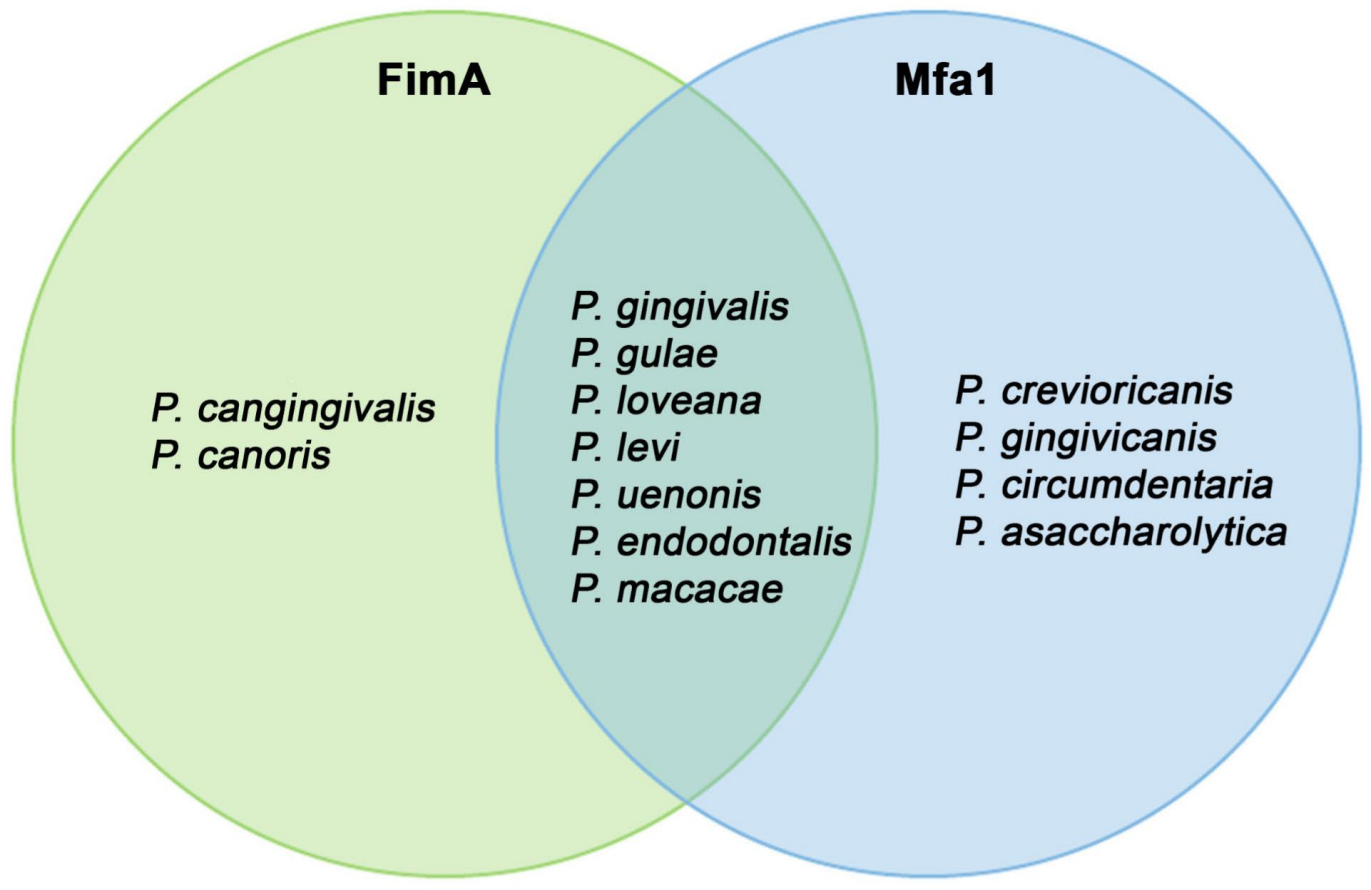
Organisms with FimA and MfaA observed in the *Porphyromonas* genome dataset. This Venn diagram showed which organisms contained encoded both phylogenetically-verified FimA and MfaA, and which organism contains Mfa1 or FimA only.

Organisms with detected FimA and Mfa1 homologs can be grouped (Figure 10). More species harbored Mfa1 compared to FimA. Two species, *P. cangingivalis* and *P. canoris*, contained FimA but not Mfa1, and four organisms (*P. crevioricanis, P. gingivicanis, P. circumdentaria,* and *P. asaccharolytica*) contained Mfa1 and not FimA. *P. crevioricanis* and *P. gingivicanis* were isolated from the gingival crevicular fluids of dogs (Hirasawa and Takada, 1994), whereas *P. circumdentaria* and *P. asaccharolytica* were isolated from the gingival margins of cats (Love et al., 1992), and from a variety of nonoral clinical samples (Shah and Collins, 1988). These differences in gene content may imply that those organisms evolved to contain the minor fimbria to function in other roles outside of the periodontal disease. A more profound study can help to elucidate the properties of those Mfa1 homologs.

The evolutionary patterns of the hemagglutinin-containing proteins are more straightforward. The phylogenetic compartmentalization between arginine-gingipains (RgpA), lysine-gingipains (Kgp), and hemaglutinins are clear. Interestingly, this tree only included proteins from the trifecta *P. gingivalis - P. gulae - P. loveana.* Gingipains were found only in *P. gingivalis* and in very few strains of *P. gulae*, whereas hemagglutinins were found in the three related organisms. The tree divided the Lys-gingipains into two lineages, associated with HG66 and W83 strains, respectively. The HG66 is an avirulent *P. gingivalis* strain exhibiting the type I genotype FimA, whereas the strain W83 is a more virulent variant, exhibiting the type IV genotype FimA (Mendez et al., 2019). The divergence between both lineages may be associated with differences in the virulence of the *P. gingivalis* strains, as a signal of a more specific adaptation to human host as a disrupting virulence factor. On the other hand, hemaglutinins (HagA or HagB) are well conserved in the trifecta, as a virulence factor important for heme acquisition, periodontal inflammation, and tissue breakdown. The evolutionary pattern of those hemaglutinins is almost completely species-specific, suggesting a strong vertical transference pattern, and potentially, another host-specific adaptation.

## 5. Conclusion

The following study, aimed to understand the evolution of *P. gingivalis* in the context of the *Porphyromonas* genus, could confirm the high relatedness with *P. gulae* but also found an unexpected relatedness with the marsupial-associated *P. loveana*; in coincidence with this relationship, several marker genes are more conserved in this group in comparison with the rest of Porphyromonads, suggesting that this group of three organisms was involved in the origin of the phenotype causing periodontal disease. Since those organisms have a characteristic presence in mammals, periodontal disease could be as old as the origin of the mammals.

The phylogenomic approach also found an interesting set of described and undescribed species, offering new opportunities for posterior studies. The phylogenetic analysis of the markers also opens the opportunity to study the properties of those virulence factors in the non-periodontal *Porphyromonas* species.

## Data availability statement

The datasets presented in this study can be found in online repositories. The names of the repository or repositories and accession number(s) can be found in the article / Supplementary material.

## Author contributions

JC and CC conceived the study. MM-O, JN-B, DG, and JC analyzed the data. MM-O, JN-B, DG, EV, JR-P, CC, and JC collaboratively elaborated, edited, and corrected the text and figures in the manuscript. All authors contributed to the article and approved the submitted version.

## Funding

JC is supported by ANID Fondecyt project 11200209, CC is supported by ANID Fondecyt project 11190073, and JR-P is supported by ANID Fondecyt project 1221064. JN-B is supported by the Integrative Genomics Ph.D. program (Universidad Mayor, Chile).

## Conflict of interest

The authors declare that the research was conducted in the absence of any commercial or financial relationships that could be construed as a potential conflict of interest.

## Publisher’s note

All claims expressed in this article are solely those of the authors and do not necessarily represent those of their affiliated organizations, or those of the publisher, the editors and the reviewers. Any product that may be evaluated in this article, or claim that may be made by its manufacturer, is not guaranteed or endorsed by the publisher.

## Footnotes

^1^https://github.com/rambaut/figtree

^2^https://github.com/SioStef/panplots/

^3^https://cran.r-project.org/web/packages/pegas/index.html
