# Supplementary figures and images for: Phylogenomic analysis of the Porphyromonas gingivalis - Porphyromonas gulae duo: approaches to the origin of periodontitis
Source: Front Microbiol. 2023 Jul 19;14:1226166. doi: 10.3389/fmicb.2023.1226166 (PMC10394638; doi:10.3389/fmicb.2023.1226166)

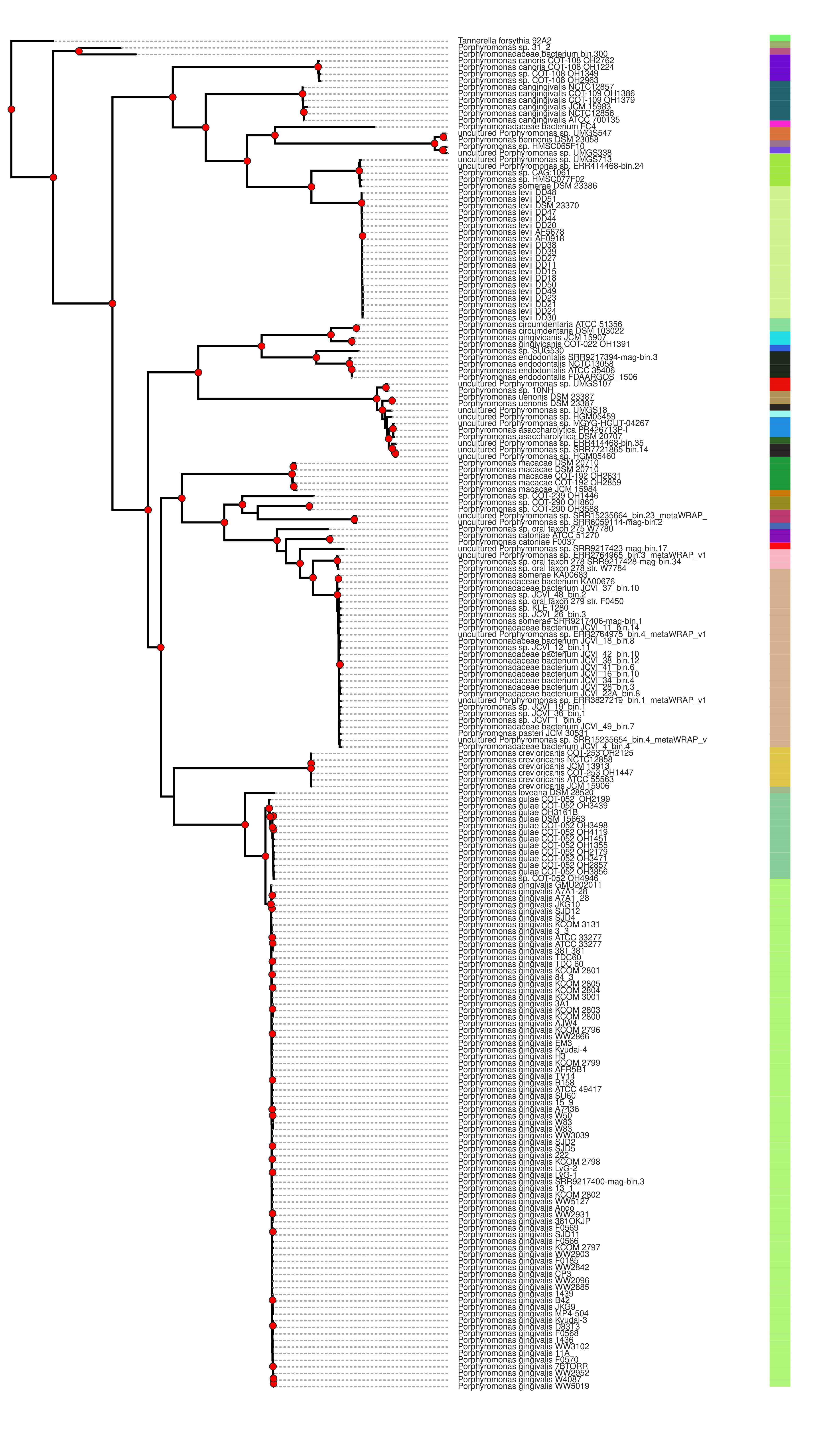

Supplement: Supplementary file 1 [file Image_1.tiff]

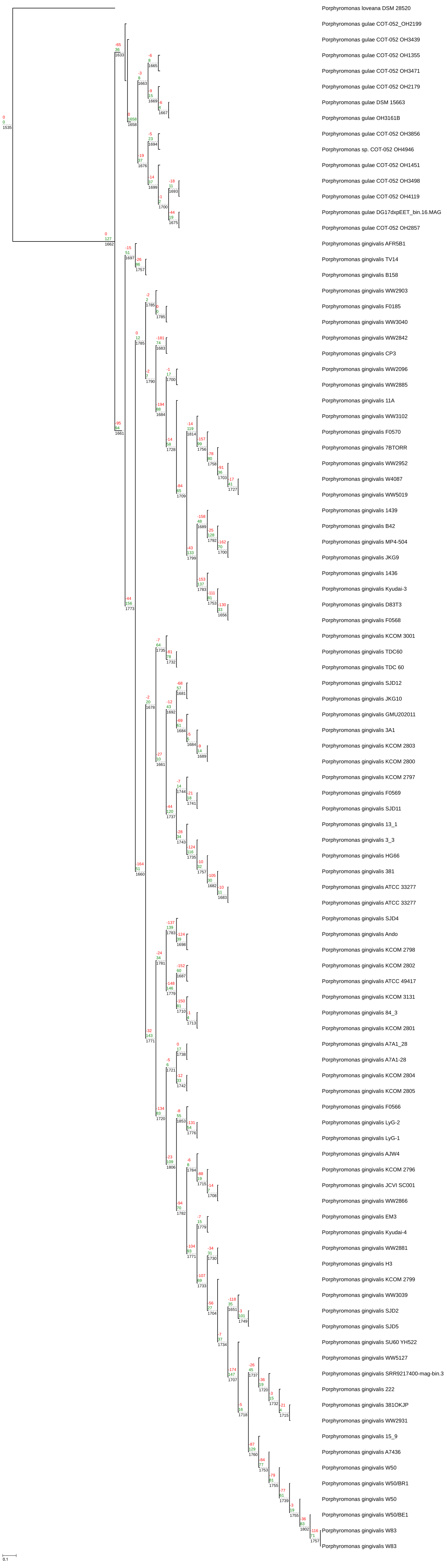

Supplement: Supplementary file 2 [file Image_2.tiff]

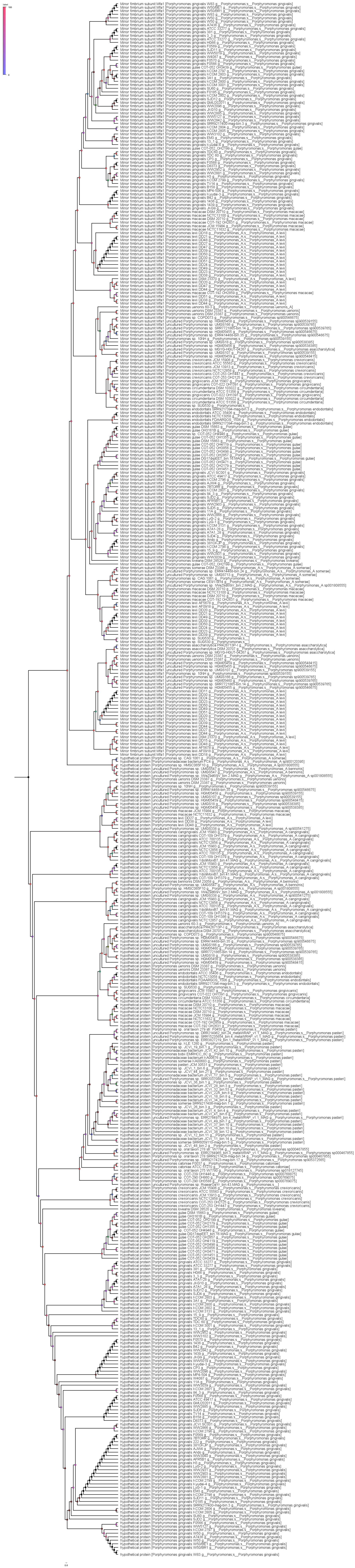

Supplement: Supplementary file 4 [file Image_4.tiff]
